# Localized Charge on Surfactant-Wrapped Single-Walled Carbon Nanotubes

**DOI:** 10.1021/acs.jpclett.2c02650

**Published:** 2022-11-11

**Authors:** Erin E. Christensen, Mitesh Amin, Trevor M. Tumiel, Todd D. Krauss

**Affiliations:** ^†^Department of Chemistry and ^‡^The Institute of Optics, University of Rochester, Hutchison Hall, Box 270216, Rochester, New York14627, United States

## Abstract

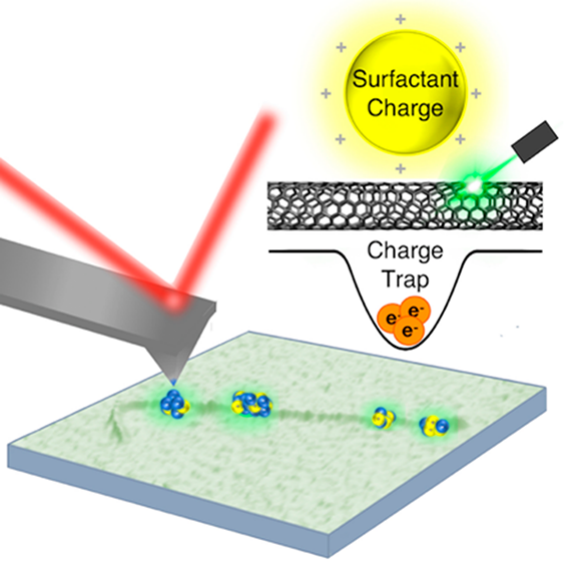

As-synthesized, semiconducting single-walled carbon nanotubes (SWCNTs) are nominally charge neutral. However, ionic surfactants that are commonly used to disperse SWCNTs in solution can lead to significantly charged aggregates adsorbed to the nanotube. Here, electrostatic force microscopy (EFM) was used to characterize the static-charge interactions between individual SWCNTs and the local environment. We report nonuniform spatial charge distributions with highly varying magnitudes ranging between ±15 *e* associated with surfactant coverage on long SWCNTs (>1.5 μm). EFM images acquired after resonant photoexcitation demonstrate charge carrier localization due to electrostatic interactions with charged surfactant aggregates. Charge densities as measured by EFM are used to estimate the depth of this electrostatically induced potential well, calculated to be on the order of hundreds of millielectronvolts, suggesting that surfactant charges heterogeneously covering SWCNTs provide traps for excitons potentially leading to their localization.

Single-walled carbon nanotubes (SWCNTs) are quasi-one-dimensional graphitic materials with unique size-dependent optical and electronic properties,^1^ making them potentially suitable for a variety of applications, including water oxidation,^2,3^ energy storage,^4^ photovoltaics,^5^ field-effect transistors,^6,7^ chemical and biological sensors,^8−13^ and quantum technology.^14^ Their relevance for these applications is owed to their remarkable physical properties, including ultrahigh mechanical strength,^15^ outstanding electrical conductivity,^16,17^ and infrared optical activity,^18−20^ all of which fundamentally result from the strength of the carbon–carbon bond and the effects of quantum confinement around the nanotube circumference.

Certain applications require well-isolated individual SWCNTs; however, SWCNT fabrication techniques yield a heterogeneous assortment of chiralities and lengths that inherently aggregate into bundles due to the strong van der Waals interactions between nanotube sidewalls.^21^ Techniques for separating bundles of SWCNTs allowed for some exploration of their photophysical properties,^22,23^ but their insolubility in both water and organic solvents proved to be a significant problem. Dispersing SWCNTs by employing either covalent^24,25^ or noncovalent^26^ methods is a useful way to increase the chemical compatibility of nanotubes with the target medium and to minimize their tendencies to aggregate into bundles. Noncovalent functionalization, based on interactions such as the physical adsorption of surfactants and polymers on the SWCNT surface,^26−32^ is advantageous as it preserves the integrity of nanotubes.^33,34^ Thus, significant attention has been directed to the study of suspending SWCNTs using surfactants, polymers, and biological molecules, all of which effectively facilitate dispersions of individual SWCNTs.

Although as-synthesized SWCNTs are nominally neutral, some studies have indirectly inferred the presence of a significant surface charge on nanotubes.^35−37^ For example, counterions adsorbed to the nanotube surface lead to localized charges along the SWCNT^38^ which can trap excitons upon photoexcitation^39^ and thus lead to single photon emission characteristics. However, the spatial location and magnitude of such charges in SWCNTs are largely unknown, and a clear understanding of the relationship between these charges and exciton localization is lacking.

Considering the common suspension of SWCNTs into a useful state is often with ionic surfactants, a study of the electrostatic interactions between an individual nanotube and its surrounding environment is crucial. Here, we image the spatial location and quantify the magnitude of surfactant charges adsorbed to SWCNTs with electrostatic force microscopy (EFM). We show that surfactant coverage on SWCNTs yields surprisingly large charge density variations with magnitudes on the order of ±15 *e* per EFM spatial resolution of 2500 nm^2^. After resonant photoexcitation, EFM measures significant charge fluctuations along the nanotube due to an electrostatic interaction between charged surfactant aggregates and photoexcited free carriers, leading to their localization. Our findings indicate that surfactant charge impurities significantly warp the local potential energy of the SWCNT, similar to sp^3^ defects which have demonstrated to be excellent single-photon emitters for quantum information science.^40,41^

EFM allows for a quantitative determination and imaging of localized charges and dielectric constants at the nanometer scale.^42−47^ In our EFM experiment (Figure S1), a first pass of the cantilever measures the topography of the sample. In a subsequent second pass, the tip is lifted off the surface and scanned at a constant height under an applied AC and DC voltage and is thereby sensitive to long-range capacitive and Coulombic forces.^45,48^ By modeling the surface charge–cantilever force through a Coulombic interaction, quantitative determinations of surface charge magnitude and location are possible.^42−44^ By modeling the cantilever–substrate force as a capacitive interaction, local variations in dielectric constant (which affect the tip–substrate capacitance) can be determined quantitatively^45^ (see the Supporting Information for a detailed description of EFM).

A typical set of EFM images is shown in Figure 1 for a long SWCNT spun coat onto a Si^++^ substrate coated with a few nanometers thick oxide layer. Figure 1a shows a topographic map of the sample surface from which we can extract the diameter and length of an individual SWCNT. The image in Figure 1b corresponds to the raw and unprocessed changes in cantilever resonance frequency at the first harmonic of the AC voltage, , from which both linear and areal charge densities are calculated for thin surfactant coated regions along the nanotube and large surfactant aggregates, respectively (see the Supporting Information for charge modeling details). The corresponding charge profile image (Figure 1d) is in units of electron charge with a spatially averaged pixel resolution of 13 nm × 13 nm. A white dashed line tracing the nanotube is overlaid onto the charge profile image to indicate the location of the SWCNT. The image in Figure 1c corresponds to changes in the cantilever resonance frequency at the second harmonic of the AC voltage, , from which we determine the local dielectric constant. For the dielectric contrast image, the SWCNT will show up brighter than the background regardless of the charge due to the larger dielectric constant of the SWCNT compared to its surroundings. While the topographic and dielectric images show a homogeneous SWCNT along its entire 2 μm length, (Figures 1a,c), the EFM charge profile image reveals heterogeneity in static charge along the SWCNT (Figure 1d). For the dozens of long SWCNTs studied with length greater than 1.5 μm, we found charge variations from surfactant coverage all along the nanotube with magnitudes on the order of 1 *e* to 15 *e*, with both positive and negative signs. The sign convention for charge densities used in this study is as follows: positive areas (yellow) corresponds to positive charge, and negative areas (blue) correspond to negative charge.

**Figure 1:**
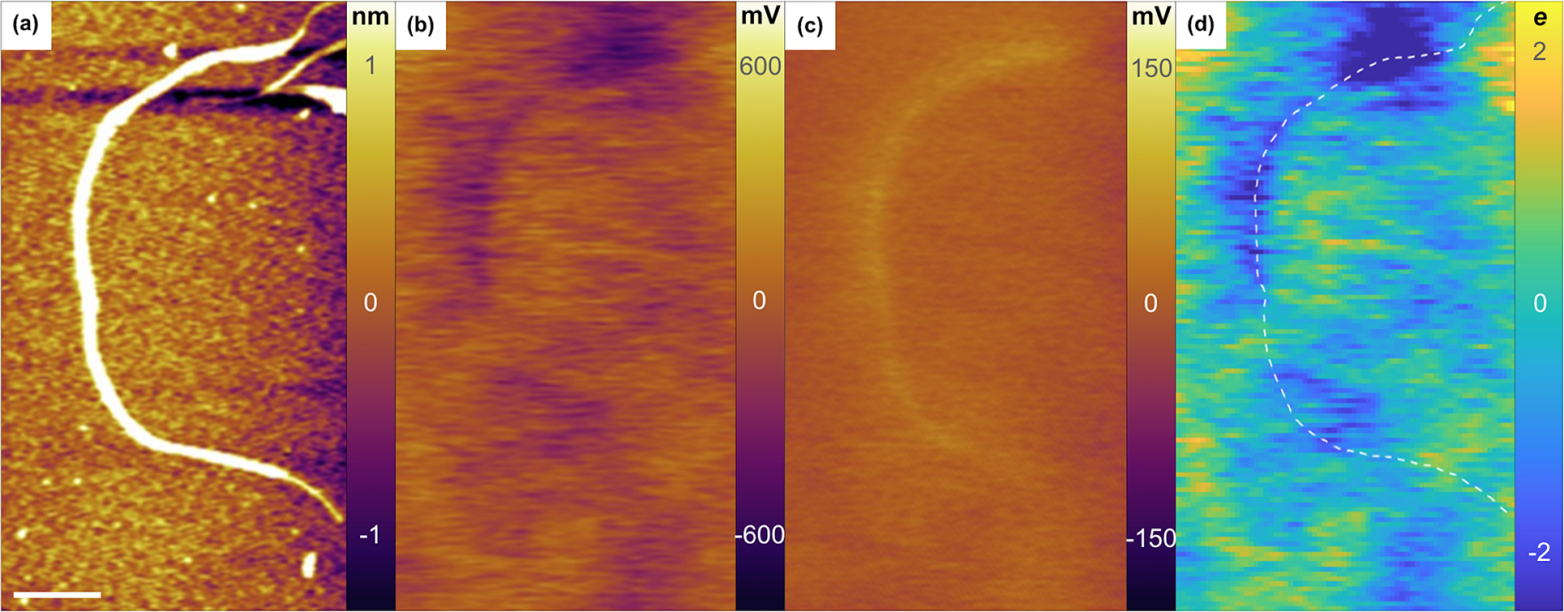
EFM images of (a) topography, (b) cantilever response at , (c) cantilever response at , and (d) charge profile image in units of electron charge with a spatially averaged pixel resolution of 13 nm × 13 nm (see the Supporting Information for conversion voltage image (b) to charge). The white dashed line is a trace of the nanotube to guide the eye. Scale bar is 200 nm.

Our EFM data are consistent with prior reports showing that ionic surfactants can form structureless aggregates on the SWCNT surface.^49^ In general, we found that both the size and charge of surfactant aggregates vary considerably (see Figure S2 for a histogram showing a distribution of the measured size and charge of surfactant aggregates). For example, the EFM charge profile image of a sodium cholate (SC)-coated SWCNT shows nonuniform charges and a nonuniform surfactant coating along the length of the nanotube (Figure 2a,b). This particular SC-coated SWCNT has calculated linear charge density values ranging between −160 *e*/m and 40 *e*/m (or, equivalently, −8 and +2 *e* per diameter of the EFM tip) with the higher magnitudes of charge originating from regions of heavier surfactant coverage. Likewise, EFM measurements of surfactant aggregates away from the SWCNT show both positive and negative charges of similar magnitude to what we observe on the surfactant coated SWCNT (white circle, Figure 2a,b).

**Figure 2:**
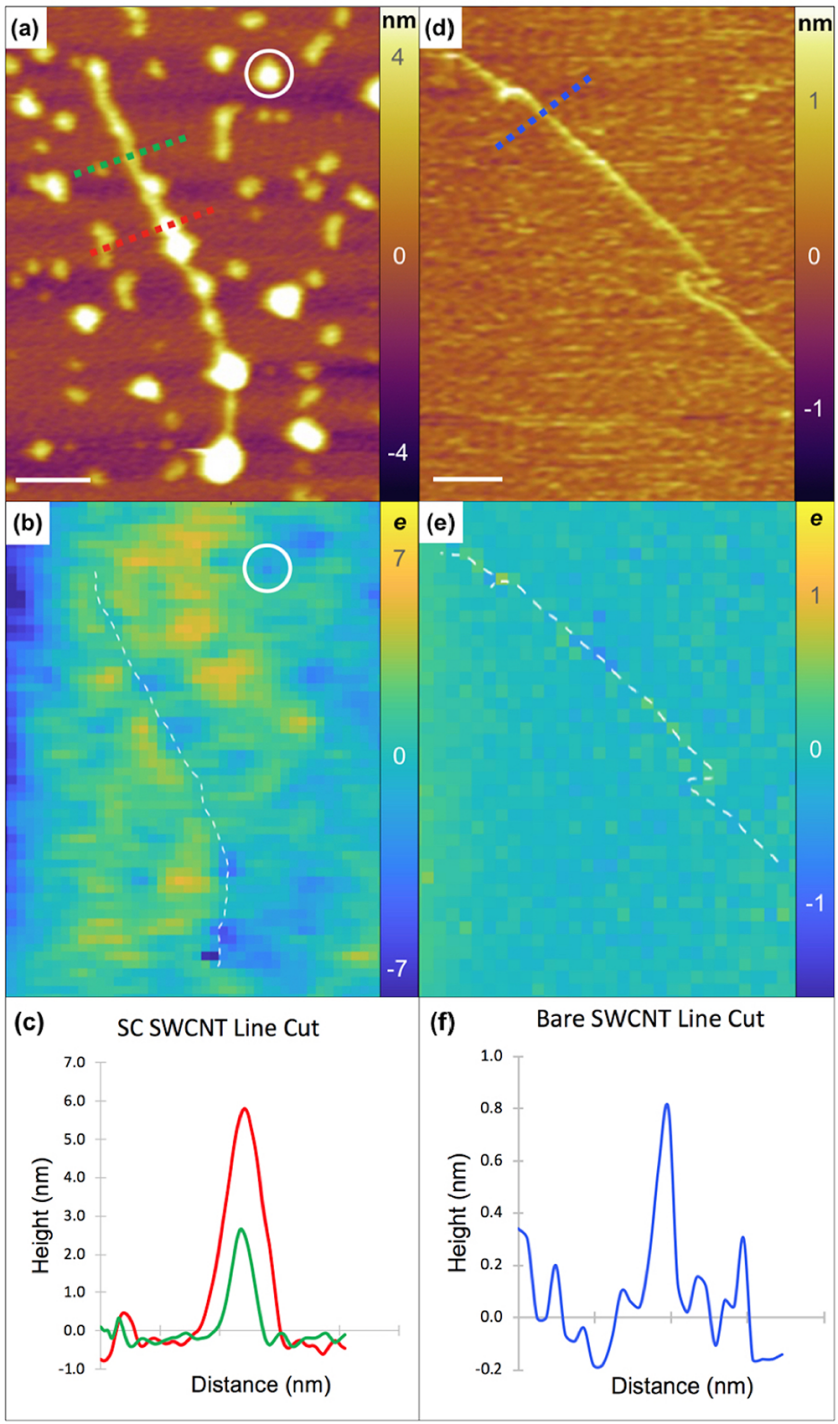
(a) Topographic image of a SWCNT coated in surfactant. (b) Calculated EFM charge profile image for the coated SWCNT in units of electron charge with a spatially averaged pixel resolution of 27 nm × 27 nm. The white dashed line is a trace of the nanotube to guide the eye. (c) Line cuts along the red and green dashed lines from the topographic image. The red line cut measures ∼6 nm in height. The green line cut measures ∼3 nm in height. (d) Topographic image of a bare SWCNT that was rinsed with IPA to remove DOC surfactant. (e) Calculated EFM charge profile image for the bare SWCNT in units of electron charge with a spatially averaged pixel resolution of 48 nm × 48 nm. (f) Line cut along the blue dashed line from the topographic image. The blue line cut measures ∼0.8 nm height indicating the diameter of a bare (6,5) SWCNT. Scale bars are 200 nm.

To confirm that the measured charges were originating from the surfactant and were not associated with the SWCNT, artifacts of the EFM system, ambient conditions, or the Si^++^ substrate, we repeated the measurements with another ionic surfactant, sodium deoxycholate (DOC), which could be efficiently rinsed off of the SWCNTs. Initially, the DOC-wrapped SWCNTs produced comparable charges to the SC-wrapped SWCNTs. Once the surfactant was rinsed off of the SWCNT (Figure 2d), the EFM charge profile image of the uncoated SWCNT became significantly more homogeneous (Figure 2e), with the SWCNT imaging approximately neutral and calculated charge values never exceeding a fraction of an electron charge (per resolved area of the EFM tip). To further confirm the surfactant was completely washed off, we generated a height profile by making a line cut across the SWCNT in the AFM topographic image (Figure 2d). We found that the bare SWCNT had a height of approximately 0.8 nm (Figure 2f), which is consistent with the diameter of a (6,5) SWCNT.^50^ AFM and EFM measurements alone cannot experimentally distinguish between certain small diameter chiralities of SWCNTs. However, CoMoCAT carbon nanotubes are majority (6,5) chirality after suspension (Figure S3). On the basis of that prevalence, we assume the (6,5) chirality for SWCNTs in various experimental and theoretical analyses throughout this study. We measured the heights of the unrinsed SWCNT (Figure 2a) at regions of light (green line cut) and heavy (red line cut) surfactant coverage. The measured diameters were approximately 3 and 6 nm (Figure 2c), respectively, indicating a substantial surfactant coating that is responsible for the measured charge. To ensure the measured charge was not impacted by the presence of water, an additional control experiment was performed. A sample of SC-SWCNT was annealed prior to imaging under nitrogen flow. EFM signals (Figure S4) acquired after heating resulted in similar charge magnitudes as unheated samples, thus confirming that any water molecules intercalated within the tubes or surfactant aggregates (in unheated samples) do not lead to any measurable charge screening.

Our EFM measurements show that surfactant aggregates are charged; however, what remains unclear is the extent of the impact these adsorbed charges have on the local energy band structure of the SWCNT. To address this question, we performed EFM measurements of surfactant-coated SWCNTs before and after photoexcitation at 561 nm (*E*_22_ transition in (6,5) SWCNTs). Figure 3a shows the topographic image of a photoexcited SWCNT on the Si^++^ substrate (with a few nanometers of oxide). Figure 3b displays the EFM charge profile image measured before photoexcitation. Large surfactant aggregate regions of the nanotube have a much greater charge magnitude compared to lightly coated regions, with areal surface charge densities upward of 6000 *e*/m^2^ (∼+15 *e* per the area of the EFM tip). Meanwhile for lightly coated regions of the nanotube, EFM measures linear charge densities fluctuating anywhere between ±100 *e*/m (±5 *e* per diameter of the EFM tip).

**Figure 3:**
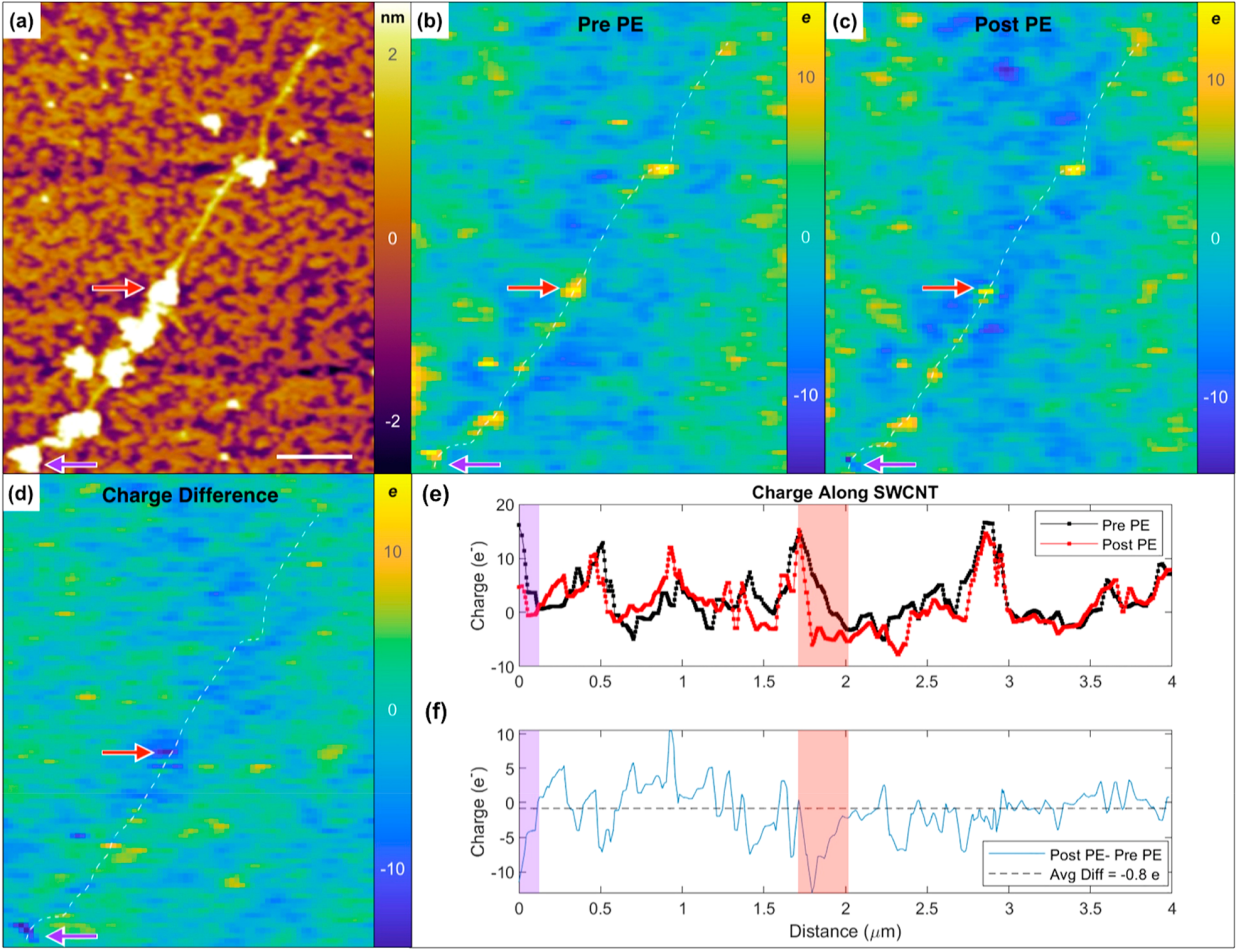
(a) EFM topography and calculated charge profile images (b) before and (c) after 561 nm wavelength photoexcitation for an individual nanotube covered in sodium cholate surfactant. (d) Charge difference image highlighting the change in charge after photoexcitation ((c) minus (b)). Charge profile images are in units of electron charge with a spatially averaged pixel resolution of 37 nm × 37 nm. Scale bar is 500 nm. (e) Plotted charge along the length of the SWCNT before (black) and after (red) photoexcitation. (f) Plotted charge difference (post PE minus pre PE) along the length of the SWCNT. Purple and red shaded regions correspond to arrow locations in (a–d). The dashed black line is the average charge of the charge difference plot.

Given that the Fermi level of Si^++^ lies squarely in the bandgap of a (6,5) SWCNT,^51,52^ we expected no preferential increase in charge magnitude or a change in charge sign upon photoexcitation. In fact, we expected that the photoexcitation of highly mobile excitons on the SWCNT would lead to a partial neutralization of static charge signals. EFM measurements after photoexcitation indicate the charged surfactant aggregates on the nanotube surface create local electrostatic perturbations that lead to distinct localized regions of charge. For example, as seen in the charge difference image (Figure 3d), photoexcitation resulted in a portion of the surfactant–SWCNT system (i.e., red arrow in Figure 3d and red shaded region in Figures 3e,f) to become more negative by ∼14 *e*. Likewise, another portion of the surfactant–SWCNT system (i.e., purple arrow in Figure 3d and purple shaded region in Figure 3e,f) becomes ∼12 *e* more negative. These localized regions of negative charge are likely due to preferential trapping of the photoexcited electron due to a change in the SWCNT’s local potential energy at the positively charged surfactant aggregate. Likewise, as demonstrated in Figure S5, we also measure hole trapping at negatively charged surfactant aggregates, showing that localized regions of negative charge can attract photoexcited holes. The EFM-measured charge profile of the surfactant–SWCNT system should not change unless there is an electrostatic interaction between charged surfactant aggregates and photoexcited free charge carriers within the SWCNT. If the surfactant charge is not altering the SWCNT local potential energy, the photoexcited charge carriers would delocalize throughout the length of the nanotube and charge profile should remain the same after photoexcitation. There are slight changes in background surfactant charge after photoexcitation, potentially due to small SWCNT fragments hidden by the aggregate and/or heat induced surfactant rearrangements during photoexcitation (see Figure S6 for background surfactant aggregate charge statistics). Ultimately, the changes in charge (50% of aggregates changing by less than 1 *e*, 90% of aggregates changing by less than 3 *e*) are much smaller than the photoinduced changes at the localization sites measured along the SWCNT which vary by magnitudes of ∼10 to 20 *e*.

We also sought to understand whether photoexcitation led to photoionization of the SWCNT or a redistribution of the free electron and hole carriers along the SWCNT. To address this question, we calculated the net charge across the nanotube as shown in Figure 3. We found the charge integrated over the entire nanotube after photoexcitation only decreases by 0.8 *e*, strongly suggesting photoexcited charge carriers are not preferentially transferred to the substrate. The fact that the total charge of the nanotube remains relatively unchanged after photoexcitation implies that the large, local changes in charge (such as at the arrows in Figure 3) are compensated elsewhere in the nanotube. Thus, photoexcitation of the SWCNT on the substrate leads to an overall charge redistribution rather than a preferential photoionization.

The degree of charge localization due to the electrostatic potential barriers caused by surfactant aggregates on the nanotube surface can be estimated by a simplified point charge model (see the Supporting Information for a model description).^39^Figure 4c shows the parameters of the model for a positively charged surfactant aggregate (red) with a charge density of 3400 electrons/μm^2^ equivalent to the measured 8.5 *e* charge per 2500 nm^2^ pixel as shown in Figure 4b. The calculated Coulombic interaction between the surfactant area and the electron on the (6,5) nanotube surface results in an overall potential energy distribution given by Figure 4d. Solving the one-dimensional Schrödinger equation for an electron trapped in the potential well of Figure 4d results in a ground state binding energy of about −0.4 eV with the electron localization width of about 70 nm (Figure 4e) along the length of the nanotube. While this purely 1D model likely overestimates the energy well depth, nonetheless, it suggests that upon photoexcitation photoexcited charges have the possibility to trap along the charged surfactant regions of the nanotube.

**Figure 4:**
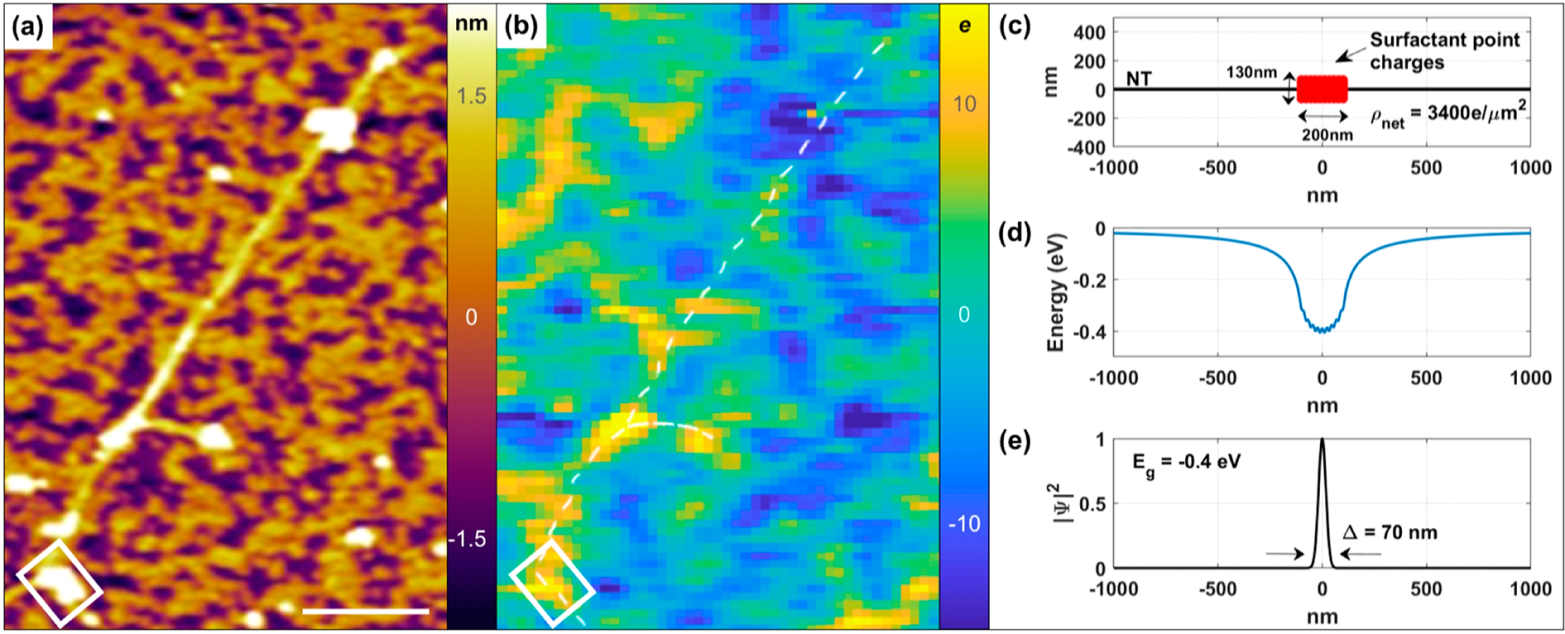
EFM images of (a) topography and (b) charge profile before photoexcitation for a SWCNT coated with sodium cholate surfactant. The charge profile image is in units of electron charge with a spatially averaged pixel resolution of 35 nm × 35 nm. The white rectangle in (a) and (b) surrounds a surfactant aggregate whose average charge measures 8.5 *e* per the area of the EFM tip for net charge density of 3400 *e*/μm^2^ as simulated for a point charge model across a 200 nm × 130 nm area in (c). The charge magnitude scale ranges ±15 *e*. Scale bars are 1 m, (d) Coulombic potential energy between point charges and a free (photogenerated) electron on the NT surface, and (e) ground state (*n* = 1) wave function of a trapped electron with calculated localization width (1/e^2^).

From our results, we infer that regions of charge localization will undoubtedly impact SWCNT photophysical properties. Exterior charges which form potential barriers (Figure 4) can impede the free movement of excitons down the length of the nanotube, thus leading to local exciton traps. Such charge induced localization sites may result in efficient radiative recombination and thus brighter photoluminescence upon excitation. Previous reports probing exciton localization in individual SWCNTs at low temperatures have attributed sites of brighter photoluminescence regions with surface adsorbates like oxygen, nitrogen, or other environmental disorder leading to localized exciton traps.^53−55^ We hypothesize that surface charges from surfactant aggregates can also significantly contribute to the observed exciton trapping. Ultimately, these charged sites may help explain the excellent photon antibunching behavior observed at cryogenic temperatures from excitons on SWCNTs.^56^

For applications in quantum information systems, localized excitons in SWCNTs represent a novel source of single photons in the near-infrared region of the electromagnetic spectrum. Current approaches of covalently driven exciton localization, via sp^3^ chemical defects^40,41^ have shown great promise, but the covalent sidewall chemistry alters the intrinsic properties of SWCNTs and adds a myriad of photoactive states that have proven difficult to control.^57,58^ Alternatively, noncovalently driven exciton localization via controllably depositing surfactant charges or applying electric fields in the vicinity of the SWCNT could be of particular interest for quantum communication efforts because this method preserves the structural integrity of the nanotube. Our experimental data and calculations at sites of surfactant aggregates suggest warping of the potential energy could lead to localized PL in a manner reminiscent of luminescent sp^3^ defects.

In summary, we report the magnitude and sign of charge densities, as measured by EFM, associated with surfactant coverage on SWCNTs. EFM studies of individual, surfactant-coated SWCNTs demonstrate that free photoexcited carriers within the nanotube are redistributed spatially due to electrostatic interactions with charged surfactant aggregates. Our results also show that upon photoexcitation this electrostatic interaction warps the local potential energy of the SWCNT in that region, calculated to be on the order of hundreds of millielectronvolts, which hinders diffusive exciton transport and presents a possible source for potential energy fluctuations along the nanotube that localize excitons at cryogenic temperatures. The unavoidable presence of large surfactant aggregates randomly distributed along the nanotube upon drying from a suspension may lead to significant exciton trapping, even at 300 K, and thus provides a potential path to control the photophysical properties of the nanotube in ways that are highly relevant for future applications in quantum science and technology.

## Experimental Methods

### Chemicals and Substrates

CoMoCAT-manufactured carbon nanotubes (single-walled, (6,5) chirality, carbon >90%, ≥77% (carbon as SWNT)) and sodium deoxycholate (≥97%) were purchased from Sigma-Aldrich. Sodium cholate hydrate (99%) was purchased from Alfa AESAR. 2-Propanol was purchased from Thermo Fisher Scientific. Silicon wafers (with 35 Å dry thermal oxide layer) were purchased from Silicon Valley Microelectronics, Inc.

### Preparation of Suspensions Enriched with Long SWCNTs by Shear Force Mixing in Sodium Cholate

Suspensions of CoMoCAT-manufactured SWCNTS were prepared at a concentration of 0.3 mg/mL in 10 mg/mL sodium cholate in nanopure H_2_O (Barnstead Micropure, Thermo Fisher Scientific). To maintain the length of the nanotubes in the suspension, the SWCNT–surfactant solution was shear force mixed in an ice bath for 17 h (at 35 psi start point). After shear force mixing, the solution was centrifuged (Heraeus Biofuge Pico) for 1.5 h at 13000 rpm (16060*g*), and the supernatant was filtered using a 5 m pore syringe filter (Millipore Millex-SV). SWCNTs with lengths greater than 1.5 m were routinely observed during EFM data collection.

### Preparation of Suspensions of SWCNTs in Sodium Deoxycholate

Suspensions of CoMoCAT-manufactured SWCNTs were prepared at a concentration of 1 mg/mL in 5 mg/mL sodium deoxycholate (DOC) in nanopure H_2_O. The DOC-SWCNT solution was tip sonicated (Branson 450 Sonifier, 16 W, 20 kHz, constant) for 1 h while immersed in an ice bath. The resulting dispersion was ultracentrifuged at 45560*g* (Beckman Optima L-90 K, SW-41 rotor) for 4 h, and the supernatant was filtered using a 5 m syringe filter. SWCNTs with lengths of ∼1 m were routinely observed during EFM data collection.

### Sample Preparation EFM Experiments (SC-SWCNT System)

A Si^++^substrate with a few nanometers thick oxide layer was first covered in two depositions of sodium cholate by spin coating (Speedline Technologies) 30L of 1 wt % sodium cholate solution for 60 s at 3000 rpm (and repeating) to help with SWCNT adhesion. A 2× dilution of the shear force mixed SWCNTs dispersed in sodium cholate was bath sonicated for 1 min, and then 50 L was spin-coated for 60 s at 3000 rpm. For the annealing control procedure only, the sample was heated at 110 °C for 1 h while the EFM chamber was filled with nitrogen. The sample was subsequently transferred to the EFM chamber to cool under nitrogen for 20 min. EFM measurements were performed under constant nitrogen flow.

### Sample Preparation EFM Experiments (DOC-SWCNT System)

A Si^++^ substrate Si^++^ substrate with a few nanometers thick oxide layer was first covered in two depositions of sodium deoxycholate by spin-coating 30 L of 1 wt % sodium deoxycholate in nanopure H_2_O for 60 s at 3000 rpm (and repeating) to help with SWCNT adhesion. A 2× dilution of the DOC SWCNT solution was bath sonicated for 1 min, and then 50 L was spin-coated for 60 s at 3000 rpm.

### Removal of DOC with Isopropyl Alcohol

The DOC-SWCNT covered Si^++^ substrate was soaked in isopropyl alcohol for 5 min. After removal from the IPA soak, it was further rinsed by lightly squirting IPA onto the substrate. The substrate was promptly dried under a stream of N_2_ gas and then imaged. This removal process was repeated (∼10 rounds) until AFM topographic images showed no remaining surfactant (verified visually and via line cuts of the SWCNT height).

### Electrostatic Force Microscopy Characterization

EFM measurements were performed at room temperature on an Asylum MFP-3D AFM (Oxford Instruments) using the Asylum Research ver. 12 software, modified with a DC-Offset box (homemade), a function generator (Sanford Research Systems), an oscilloscope (Tektronix), and lock-in amplifiers (EG&G) to allow for accurate measurements of charge and dielectric constants of the sample. Olympus made AC240TM-R3 silicon cantilevers with titanium–platinum coating with 5 and 20 nm thickness, respectively, and spring constants of ∼1.2–1.8 N/m from Asylum Research were used at their resonant frequencies of 62–71 kHz. Two lock-in amplifiers measured the  and  signals. Typical parameters were *V*_ac_ = 3 V peak-to-peak, *V*_dc_ = −, |*V*_dc_| < 0.5 V, = 400 Hz, lock-in time constant = 3 ms, scan rate = 0.75 Hz per line, and lift height *z* = 7–10 nm. The acquisition time for one image was ∼11 min. Charge calculations were performed by MathWorks MATLAB. Curve fitting and image analysis were performed with Igor Pro 6.3.7.2. Charge profile images were produced by MathWorks MATLAB.

### Photoexcitation Measurements

Our samples were photoexcited at a grazing angle with 561 nm laser light (CW laser) (Coherent OBIS) at an approximate intensity of 1.6 W/cm^2^. The laser was directed on the sample for 10 min, uniformly illuminating the entire nanotube under investigation. The EFM scan began immediately after the laser was turned off.

## Supporting Information Available

Details of EFM theoretical description, numerical modeling of surfactant charge, additional examples of EFM data, and statistics on charges measured (PDF)
Transparent Peer Review report available (PDF)
