# Supplementary material for: Localized Charge on Surfactant-Wrapped Single-Walled Carbon Nanotubes
Source: J Phys Chem Lett. 2022 Nov 11;13(46):10705–12. doi: 10.1021/acs.jpclett.2c02650 (PMC9706551; doi:10.1021/acs.jpclett.2c02650)
Supplement: Supplementary file 1 — jz2c02650_si_001.pdf [file jz2c02650_si_001.pdf]

Supporting Information: Localized Charge on Surfactant Wrapped Single-Walled Carbon Nanotubes

*Erin E. Christensen,[1] Mitesh Amin,[2] Trevor M. Tumiel,[1] Todd D. Krauss[1,2]**

[1]Department of Chemistry and [2]The Institute of Optics, University of Rochester, Hutchison Hall, Box 270216, Rochester, NY 14627, United States

echrist5@ur.rochester.edu, mamin@ur.rochester.edu, t.tumiel@rochester.edu, todd.krauss@rochester.edu*

Table of Contents

Description	*Page*
1. Electrostatic Force Microscopy Equations and Theory……………………………..	S2-S5
2. Distribution of Surfactant Charge and Area………………………………………….	S6
3. Absorbance Spectrum…………………………………………………………………	S7
4. Charge Profile After Annealing………………………………………………………	S7
5. Plotted Charge Along SWCNT………………………………………………………	S8
6. Background Surfactant Charge Statistics…………………………………………….	S9
7. Charge Numerical Modeling…………………………………………………………	S10-S11

1. Electrostatic Force Microscopy Equations and Theory

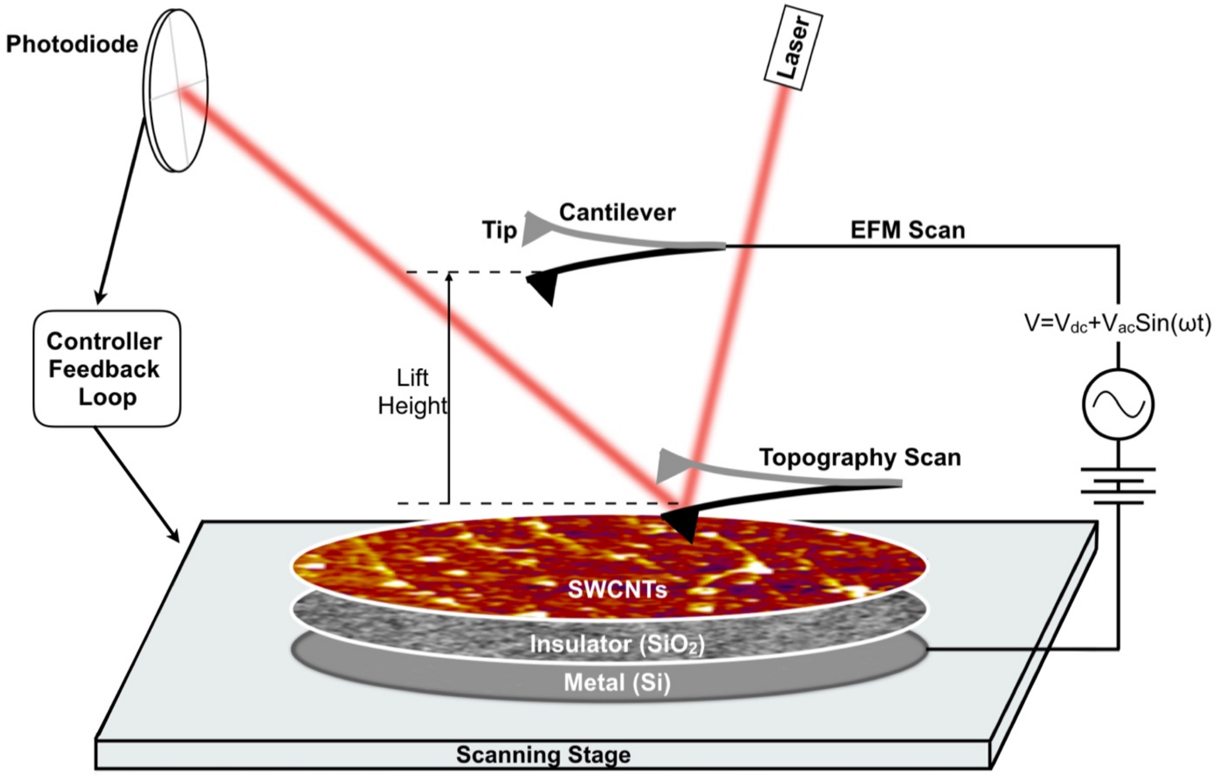

Figure S1. Schematic of the typical experimental setup for EFM. A DC (Vdc) and AC (Vac) voltage is applied to a conductive tip. Capacitive and Coulombic forces from tip-surface electrostatic interactions are detected and used to quantitatively determine dielectric constant and charge magnitude.

EFM is a modification of atomic force microscopy (AFM), which consists of an oscillating cantilever with a sharp tip at its end that scans over the sample surface. Near-range force interactions between the tip and the surface cause the cantilever to deflect. A laser is directed at the end of the cantilever and is reflected off the cantilever and directed onto a quadrature photodetector. The reflected laser signal moves according to the oscillating motion of the cantilever. The displacement of the laser signal is detected, and that signal is routed into a feedback loop that adjusts the height of the oscillating cantilever to move the deflected laser signal back to the center of the detector. The response of the cantilever in the *z*-direction is used to generate the height value for a particular point on the sample. The sample is raster scanned in the *x*-*y* direction with constant monitoring and feedback of the deflected signal, which creates a height or topography map of the sample surface.

EFM works by measuring the long-range electrostatic forces between a conductive cantilever and a conductive substrate. In this EFM experiment, a first pass of the cantilever

measures the topography of the sample. In a subsequent second pass, the tip is lifted off the surface and scanned at a constant height under an applied AC and DC voltage[1, 2]. The attractive force, *F*, between the cantilever and the substrate with applied voltage, *V*, is proportional to the square of the voltage difference between the cantilever and the substrate:

$$F = \frac{1}{2}\frac{\partial C}{\partial z}V^2 \tag{S1}$$

The forces on the EFM tip are given by capacitive and Coulombic terms.[3, 4] We model the Coulombic forces for the nanotube as an infinite 1D line charge, λ, modified from the Krauss *et al.* point charge model for CdSe nanocrystals.[1] An electrostatic attraction arises from the application of a sinusoidal voltage, $V=V_{dc}+V_{ac}sin(\omega t)$, with components of the attractive force at zero frequency, at the frequency of the applied voltage, ω, and at twice that frequency, 2ω. The force on the tip at ω, and 2ω can be determined with lock-in amplification, and written for a line charge as[5, 6]

$$F(\omega) = \frac{\partial C}{\partial z}(V_{dc} + \phi)V_{ac} + \frac{\lambda}{2\pi\epsilon_0(z+\rho)}CV_{ac} + \frac{\lambda_1}{2\pi\epsilon_0(z+\rho+{}^{2h}/_{\varepsilon_1})}CV_{ac} + \frac{\partial C}{\partial z}\frac{V_{ac}}{C}\lambda_2 D_{tip} \tag{S2}$$

$$F(2\omega) = \frac{\partial C}{\partial z}\frac{V_{ac}^2}{4} \tag{S3}$$

In the equations above, *C* is the tip-substrate capacitance, ϕ is the contact potential, ρ is the tip radius, and *z* is the separation between the insulator surface and the EFM tip. The samples consist of a p-doped silicon substrate with the surfactant dispersed nanotubes atop a thin SiO_2 insulator layer with thickness *h* and dielectric constant ε_1 . For nanotube measurements, λ is the linear charge density on the tube surface while λ_1 and λ_2 are the induced image linear charge densities on the metallic substrate and EFM tip, and assuming a parallel plate geometry between the tip and substrate as an approximation[2]

$$\lambda_1 = -\lambda\frac{z}{(z+{}^{h}/_{\varepsilon_1})} \tag{S4}$$

$$\lambda_2 = -\lambda\left(\frac{{}^{h}/_{\varepsilon_1}}{(z+{}^{h}/_{\varepsilon_1})}\right) \tag{S5}$$

Additionally, we model the large surfactant aggregates as an infinite 2D sheet charge having an areal density of σ. Typical surfactant aggregates under EFM investigation are much larger than the EFM tip diameter of 50 nm and due to the small tip height *z*, we believe an infinitely charged sheet model is a valid approximation. The surface charge density, σ, and its image interact with the charges on the EFM tip including induced surface charge due to σ. The force on the tip at *ω,* and *2ω* are written for a surface charge density as[5, 6]

$$F(\omega) = \frac{\partial C}{\partial z}(V_{dc} + \phi)V_{ac} + \frac{\sigma}{2\epsilon_0}\mathrm{C}V_{ac} + \frac{\sigma_1}{2\epsilon_0}\mathrm{C}V_{ac} + \frac{\partial C}{\partial z}\frac{V_{ac}}{C}\sigma_2 \mathrm{A_{tip}} \tag{S6}$$

$$\mathrm{F}(2\omega) = \frac{\partial \mathrm{C}}{\partial \mathrm{z}}\frac{\mathrm{V_{ac}^2}}{4} \tag{S7}$$

We note that the for the infinite 2D charged sheet, the second and third force interaction terms are independent of tip-substrate height *z* (besides the capacitance C(*z*) dependence). σ_1 and σ_2 are the induced image surface charge densities on the metallic substrate and EFM tip, and assuming a parallel plate geometry between the tip and substrate[2]

$$\sigma_1 = -\sigma\frac{z}{(z + {h}/{\varepsilon_1})} \tag{S8}$$

$$\sigma_2 = -\sigma(\frac{{h}/{\varepsilon_1}}{\left(z + {h}/{\varepsilon_1}\right)}) \tag{S9}$$

An oscillating EFM tip is modeled as a simple harmonic oscillator in a force field, so its resonant frequency, ν, is[2, 6]

$$\nu = \nu_0\sqrt{1 - \frac{1}{\kappa}\frac{\partial \mathrm{F}}{\partial \mathrm{z}}} \tag{S10}$$

where κ is the cantilever spring constant and ν_0 is the natural resonance frequency. The change in resonant frequency is

$$\Delta\nu = \frac{-\nu}{2\kappa}\frac{\partial F}{\partial z} \tag{S11}$$

With no sample present on the bare substrate, we measure the *z* dependence on $\frac{\partial^2 C}{\partial z^2}$. By holding V_{ac} and V_{dc} fixed, and measuring $\Delta\nu(\omega)$ as a function of *z*, the capacitance of the tip-substrate

system can be measured and subsequently used to determine the surface charge from the measured force on the tip at ω [2]. Taking the derivative of equation S2 with no charges present and inserting it into equation S11 gives

$$\frac{\partial^2 C}{\partial z^2} = \frac{-2\kappa}{(V_{dc} + \phi)V_{ac}} \frac{\Delta\nu(\omega)}{\nu} \tag{S12}$$

The capacitance of the tip-substrate system can be measured and subsequently used to determine the linear charge density and surface charge density, λ and σ, respectively, from the measured force gradient on the tip at ω.[7] λ and σ are multiplied by the diameter and area of the tip, respectively, to produce a charge profile image in units of electron charge. Dielectric properties can be determined by fitting the measured force on the cantilever at 2ω [6], using equation S3. In the dielectric image, $\Delta\nu(2\omega)$, there is an increase in the measured signal in the presence of a SWCNT, as expected due to the larger dielectric constant of the SWCNT compared to its surroundings. For the charge image, $\Delta\nu(\omega)$ with V_{dc} set so that $V_{dc} + \phi = 0$, three types of behavior are possible: an increase or decrease in the measured Coulombic force signal corresponding to a positive or negative charge, respectively, or $\Delta\nu(\omega) = 0$ corresponding to a neutral sample.

2. Distribution of Surfactant Charge and Area

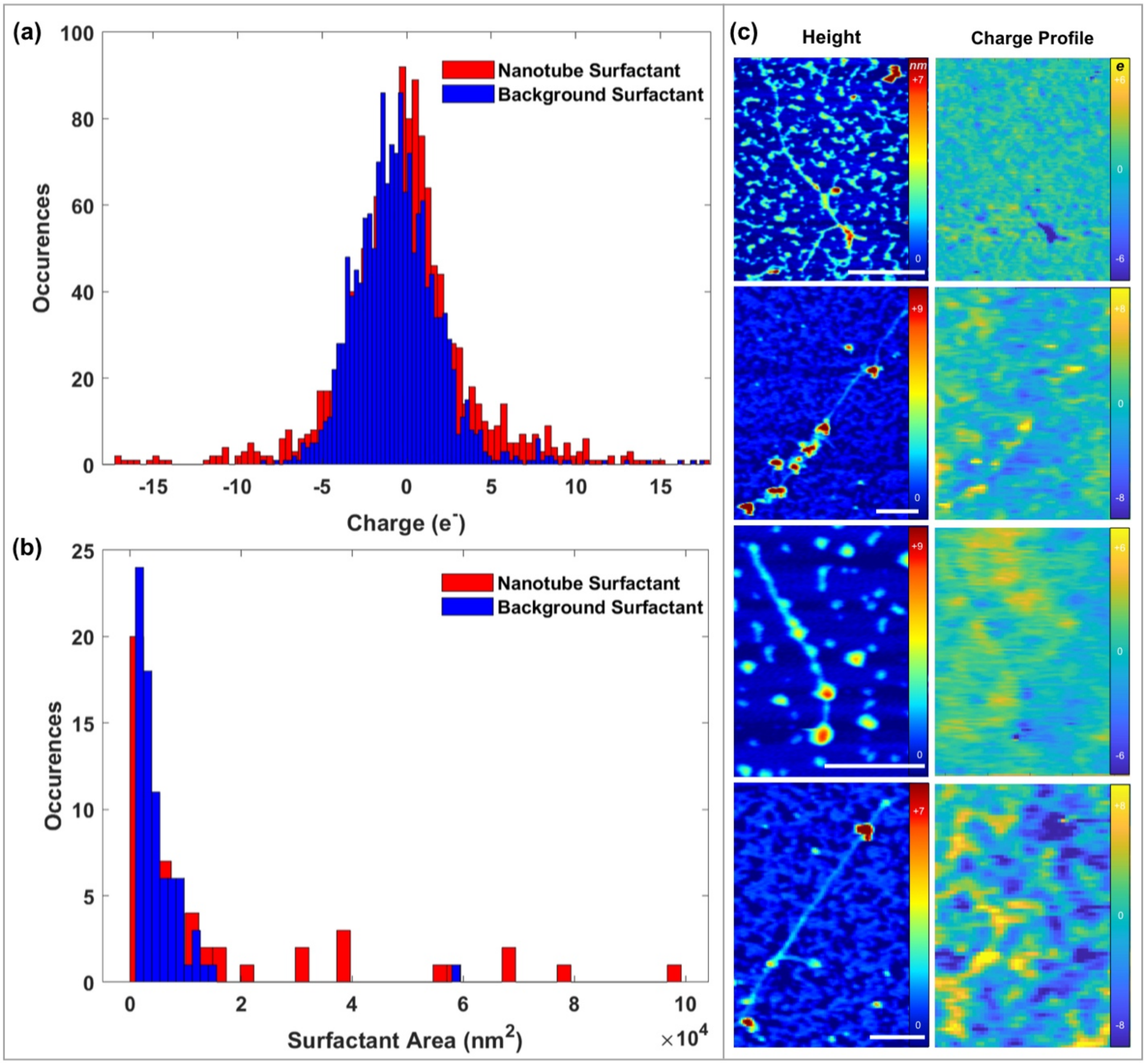

Figure S2. (a) Histogram of the measured charge values (per 50x50 nm² pixel) of surfactant aggregates on the nanotubes (red) compared to surfactant aggregates in the background of the EFM topographic images (blue). (b) Histogram of the area of surfactant aggregates on the nanotubes (red) compared to surfactant aggregates in the background of the EFM topographic images (blue). (c) Topographic (left) and corresponding charge profile (right) images from which surfactant area and charge distributions were measured. Charge profile images were generated with the infinite 2D charged sheet model to represent the surfactant charge. Scale bars are 500 nm.

3. Absorbance Spectrum

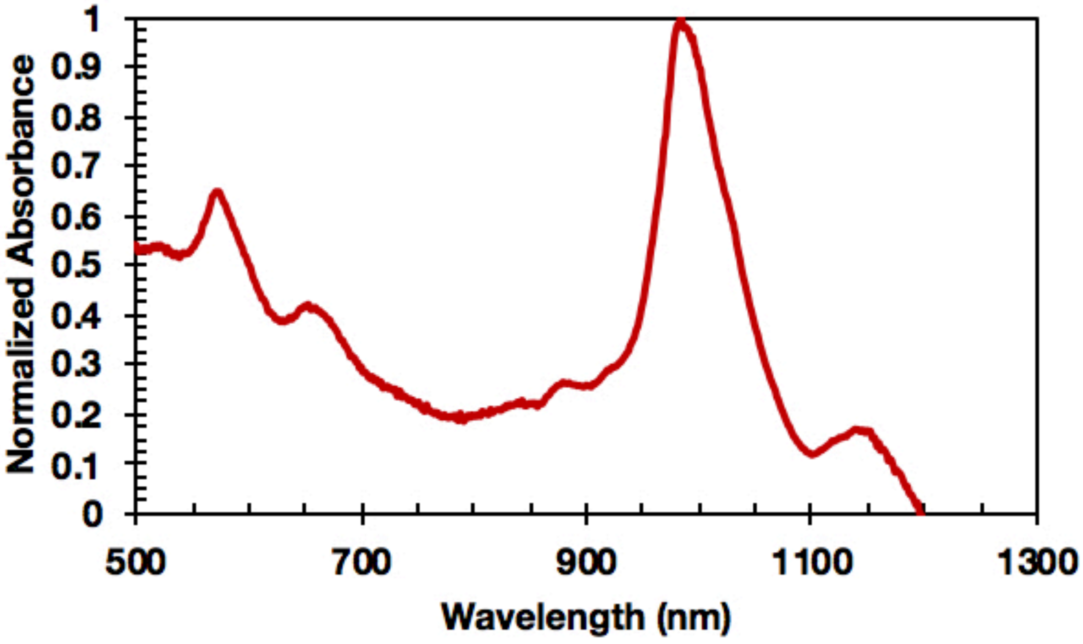

Figure S3. Normalized absorbance spectrum of CoMoCAT SWCNTs after suspension in 1% wt. sodium cholate solution. The dominant absorbance signal at ~998 nm corresponds to the E11 feature of (6,5) SWCNTs. The shoulder at ~1040 nm indicates the presence of (7,5) SWCNTs, as well.

4. Charge Profile After Annealing

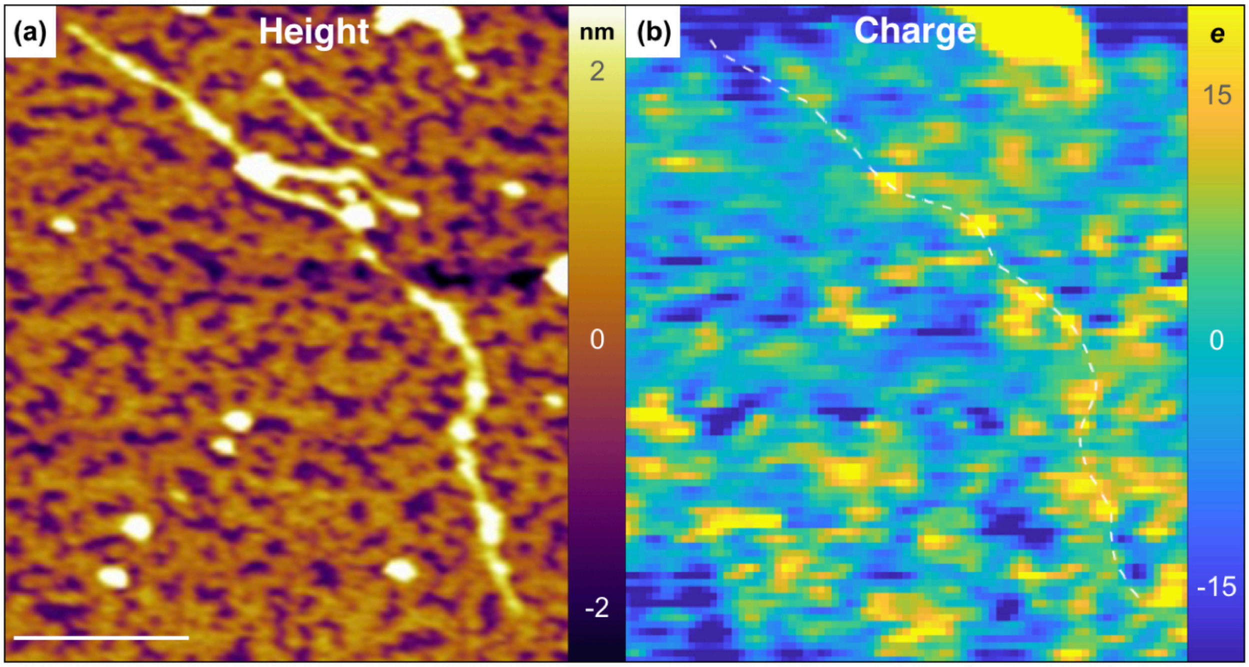

Figure S4. EFM images of (a) topography and (b) charge profile for a SWCNT coated with sodium cholate surfactant after annealing. Scale bar is 500 nm.

5. Plotted Charge Along SWCNT

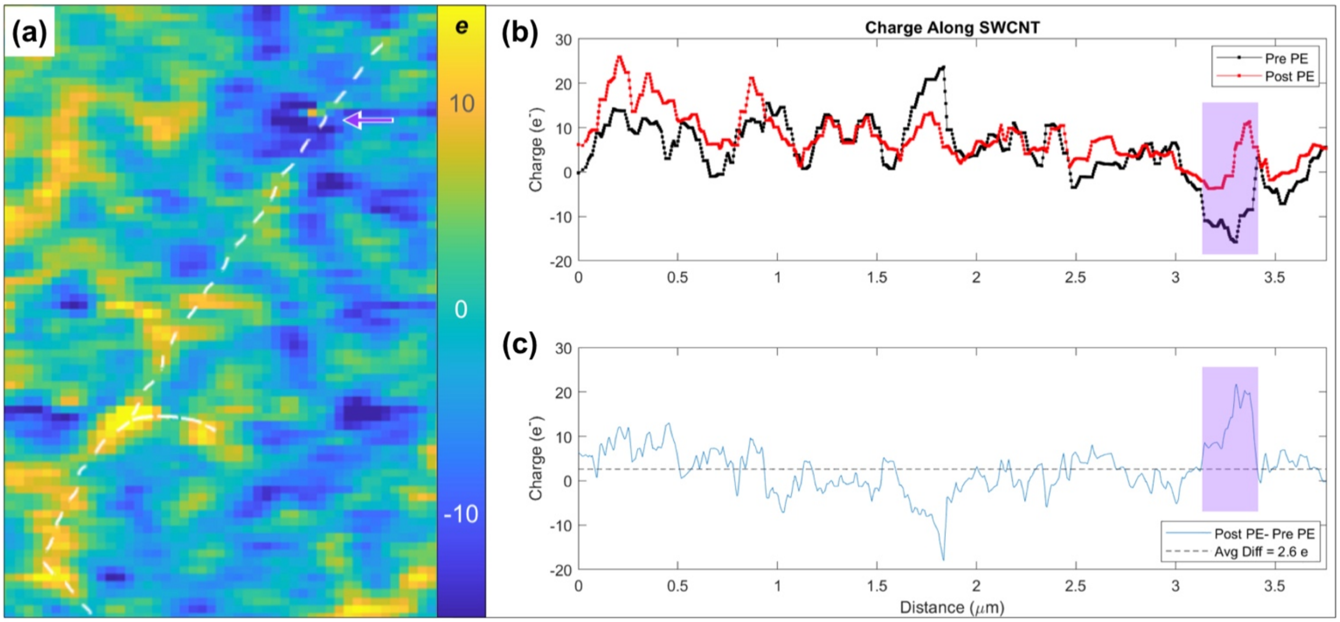

Figure S5. (a) Calculated charge profile image before photoexcitation for the nanotube in Fig. 4. Purple arrow points to a negatively charged surfactant aggregate. (b) Plotted charge along the length of the SWCNT before (black) and after (red) photoexcitation. (f) Plotted charge difference (post PE minus pre PE) along the length of the SWCNT. Purple shaded regions correspond to the arrow location in a. The dashed black line is the average charge of the charge difference plot.

6. Background Surfactant Charge Statistics

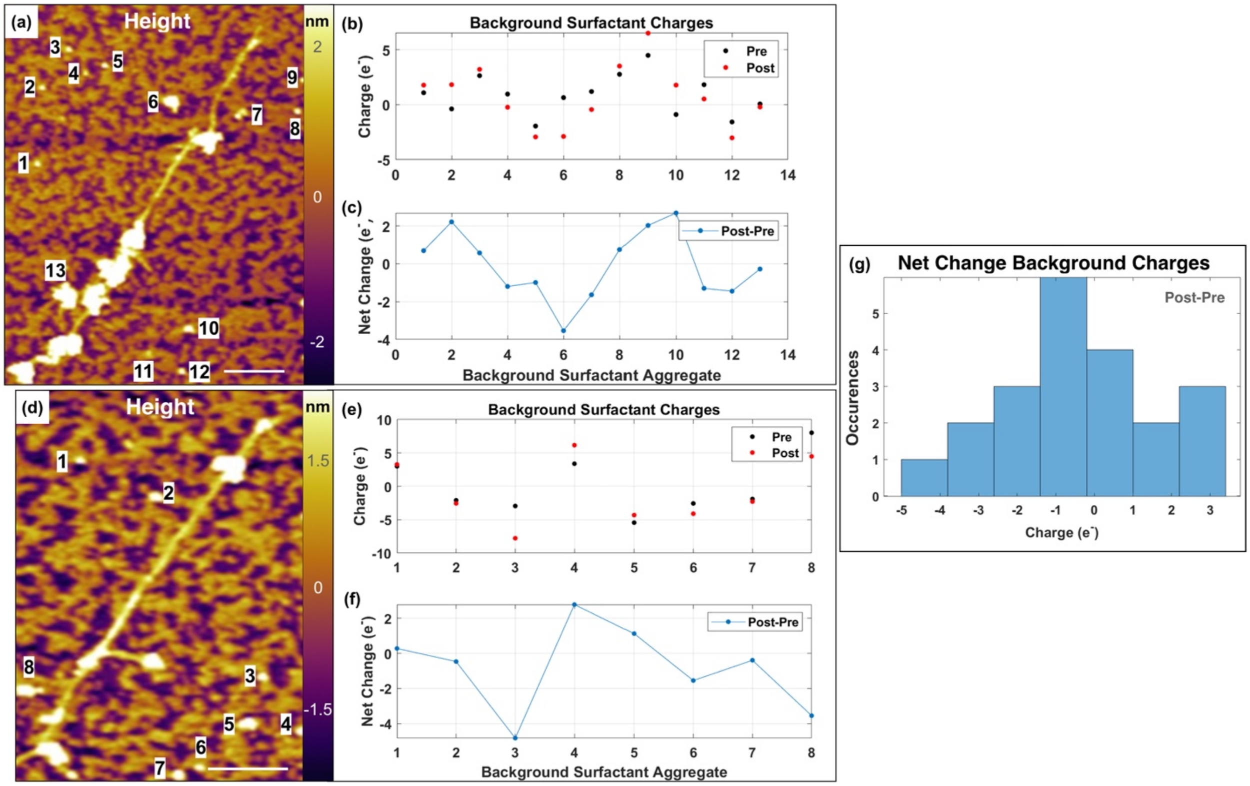

Figure S6. (a) Topographic AFM image (Figure 3) with surfactant aggregates away from the SWCNT numbered 1-13. (b) Calculated charge e pre (black) and post (red) photoexcitation plotted for surfactant aggregates 1-13. (c) Plotted charge difference (post PE minus pre PE) for surfactant aggregates 1-13. (d) Topographic AFM image (Figure 4 and Figure S5) with surfactant aggregates away from the SWCNT numbered 1-8. (e) Calculated charge e pre (black) and post (red) photoexcitation plotted for surfactant aggregates 1-8. (f) Plotted charge difference (post PE minus pre PE) for surfactant aggregates 1-8. (g) Histogram displaying the net change in charge for 21 background surfactant aggregates.

7. Charge Numerical Modeling

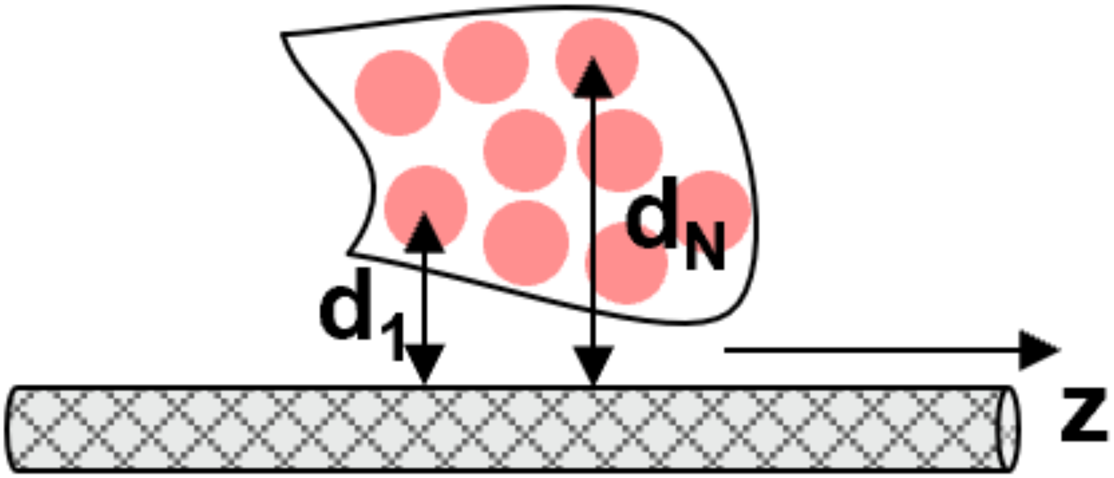

Figure S7. Schematic of the numerical modeling geometry to calculate the Coulombic interactions between a charged surfactant aggregate and a free carrier along the nanotube surface. Surfactant aggregate was divided into a point-charge numerical mesh given by red circles and individual Coulomb contributions were added to solve the 1D Schrödinger equation.

In order to estimate the extent of the electrostatic interactions between the surfactant aggregate and a free charge carrier (i.e. hole or electron) along the surface of the nanotube, a 1D Schrödinger equation was solved numerically with a Hamiltonian given by $H = \frac{-\hbar^2}{2m_{eff}}\nabla^2 + V(z)$ where the effective electron or hole mass is given by $m_{eff} = 0.07m_e$ for a (6,5) chirality SWCNT. The potential energy $V(z)$ along the axial direction of the tube as shown in Figure S7 was calculated as a summation of the individual Coulombic point charge contributions between the positive counterions in the surfactant aggregate and the freely photoexcited electrons on the tube surface:

$$V(z) = \sum_{i=1}^{N} \frac{-e^2}{4\pi\epsilon_0\epsilon}\left\{\frac{Z_i}{\sqrt{d_i^2 + (z - z_i)^2}}\right\}$$

where i represents the index of a particular point charge within the numerical surfactant charge mesh, Z is the charge valency or magnitude which is determined by the measured EFM data, d is the vertical displacement of the point charge from the tube surface, z_i is the axial displacement for the ith point charge, and $\epsilon = 6$ for the approximate relative dielectric constant of the sodium

cholate surfactant. The 1D $H\Psi_n = E_n\Psi_n$ eigenvalue problem was solved in MATLAB for the n=1 ground state as reported for an example calculation in Figure 4c-e of the manuscript.

(1) Krauss, T. D.; Brus, L. E. Charge, Polarizability, and Photoionization of Single Semiconductor Nanocrystals. *Phys. Rev. Lett.* **1999**, *83*, 4840-4843
(2) Cherniavskaya, O.; Chen, L. W.; Weng, V.; Yuditsky, L.; Brus, L. E. Quantitative Noncontact Electrostatic Force Imaging of Nanocrystal Polarizability. *J. Phys. Chem. B* **2003**, *107*, 1525-1531
(3) Martin, Y.; Abraham, D. W.; Wickramasinghe, H. K. High-Resolution Capacitance Measurement and Potentiometry by Force Microscopy. *Appl. Phys. Lett.* **1988**, *52*, 1103-1105
(4) Terris, B. D.; Stern, J. E.; Rugar, D.; Mamin, H. J. Contact Electrification Using Force Microscopy. *Phys Rev Lett* **1989**, *63*, 2669-2672
(5) Krauss, T. D.; Brus, L. E. Electronic Properties of Single Semiconductor Nanocrystals: Optical and Electrostatic Force Microscopy Measurements. *Mater. Sci. Eng., B* **2000**, *69*, 289-294
(6) Krauss, T. D.; O'Brien, S.; Brus, L. E. Charge and Photoionization Properties of Single Semiconductor Nanocrystals. *J. Phys. Chem. B* **2001**, *105*, 1725-1733
(7) Cherniavskaya, O.; Chen, L.; Islam, M. A.; Brus, L. Photoionization of Individual Cdse/Cds Core/Shell Nanocrystals on Silicon with 2-Nm Oxide Depends on Surface Band Bending. *Nano Lett.* **2003**, *3*, 497-501
