# Supplementary material for: Localized Charge on Surfactant-Wrapped Single-Walled Carbon Nanotubes
Source: J Phys Chem Lett. 2022 Nov 11;13(46):10705–12. doi: 10.1021/acs.jpclett.2c02650 (PMC9706551; doi:10.1021/acs.jpclett.2c02650)
Supplement: Supplementary file 2 — jz2c02650_si_002.pdf [file jz2c02650_si_002.pdf]

jz-2022-026509.R1

Name: Peer Review Information for "Localized Charge on Surfactant Wrapped Single-Walled Carbon Nanotubes"

First Round of Reviewer Comments

Reviewer: 1

Comments to the Author

Christensen et al. study the impact of residual surfactant on the charge state of individualized SWCNTs lying on a substrate. They use electrostatic force microscopy (EFM) to probe spatially localized charge densities that are associated with 'clumps' of surfactant, where the local charge density ranges between +/- 15 electrons. Resonant photoexcitation creates distinct localized regions of charge, suggesting the possibility that the surfactant-modified sites may provide potential traps and/or dissociation sites for photogenerated excitons. The study is done carefully and the results and conclusions are interesting, but I think that a number of questions need to be addressed before the manuscript can be published. I have several specific questions below that relate to both the qualitative and quantitative conclusions that can be drawn from the EFM measurements, as well as several questions regarding key control experiments that would shed more light on the conclusions.

- At the highest level, I'm not sure I understand how the authors confirm that the charged surfactant regions observed on SWCNTs by EFM introduce charges into the SWCNTs. The surfactant clumps show large charge density values on their own, whether or not they are adsorbed to the SWCNTs, so how does observing a charged surfactant clump on a SWCNT translate to knowledge that the observed charges are actually on the SWCNTs (i.e. the SWCNTs are doped) in that same region?
- Related to this question, I am wondering about how quantitative of a conclusion can be drawn from the EFM measurements related to the SWCNT charge density. The phrasing used in the manuscript (e.g. 'This particular SWCNT has calculated linear charge density values ranging between -160 e/um and 40 e/um…') appears to imply that all locally observed charges for a particular surfactantcoated region are assumed to be on the SWCNT. Is this the interpretation? If that is the case, can the authors use these linear charge density values to estimate what this would translate to for the Fermi level of the SWCNT? Or is the interpretation more qualitative than that, i.e. that the observation of locally charged surfactant clumps implies that the SWCNT is charged due to the presence of that local charge density, but that the total number of charges on the SWCNTs cannot be deduced from the measurements?
- The authors conclude from photoexcitation (PE) experiments that certain surfactant-coated areas of the SWCNTs become more electron- or hole-rich, potentially due to the local band structure in the coated region forming an exciton localization (or dissociation) site. Looking at Figure 3b and 3c, it looks like there are surfactant clumps off of the SWCNT that also undergo

relatively large changes in charge density after PE. How do the authors discern that the SWCNT actually plays a role in these PE experiments? Can the authors compare the spatially probed charge densities before/after PE for the area away from the SWCNT to serve as the control group to compare against? If so, what are the criteria that indicate a statistically significant change above what is observed for surfactant away from the SWCNTs?

- Water, which has a very high dielectric constant, is known to fill the endohedral space within SWCNTs, which could play an important role in the charge densities observed in the current experiments. It would also seem feasible that the large surfactant clumps that the authors observe in their experiments may have water molecules intercalated within the clumps. Can the authors comment on the degree to which water may impact their results. I also did not see any annealing step(s) discussed in the Methods. Could the authors compare the EFM-measured charge density profiles before and after an anneal step that would potentially remove some/all of the water molecules within the SWCNTs and within the surfactant clumps?
- It would be helpful for the authors to explicitly state early on the sign convention used in the charge profile images, line scans, etc. When I saw that the charge densities are provided in units of electrons, my initial interpretation (taking Figure 3 as an example) is that positive areas correspond to positive numbers of electrons (i.e. negatively charged due to electron accumulation), while negative areas correspond to regions where electrons have been removed (i.e. positively charged). It was only after reading the PE description on page 9 where the authors indicate that particular regions in Figures 3f with negative signs 'become more negative' that the authors are defining charge density in the opposite fashion of my original interpretation.
- Does the majority carrier in the silicon wafer used as the sample substrate play any role in the typical carrier type observed in the SWCNTs? In the current experiments, the authors use Si^{++} and it seems like typical pre-PE measurements indicate hole accumulation on the SWCNTs (Fig. 3e, Fig. 4b). Have the authors performed measurements on n-type Si to see if the primary carrier sign observed by EFM flips to be electron-dominated?
- Does the charge of the surfactant head group play a role in the predominant carrier type observed in the SWCNTs? Since the authors observe predominantly hole rich SWCNTs for these negatively charged surfactants, could the authors compare these measurements to a positively charged surfactant (e.g. CTAB) to tease out the impact of the head group charge?

Reviewer: 2

Comments to the Author

This report provides evidence for the localization of surfactant induced charges on SWCNTs using electrostatic force microscopy (EFM). There are many mysterious phenomena related to solution processed nanotubes; the residual surfactant is expected to play a role but surprisingly little experimental evidence exists. Using EFM, Krauss and co-workers reveal a picture that appears to make sense. This work provides valuable insights and is publishable, but I do have a few questions.

Figure 2. How is the DOC surfactant removed with IPA? This is important to understand this control, but the detail of this procedure is not provided.

Figure 3. In one of the surfactant aggregates, photoexcitation is shown to deplete the electrons, but this phenomenon is not observed in other regions of the same nanotube. Are there other factors in play here? What is the statics in observing such photoexcitation-induced depletion of electrons?

How is the (6,5) chirality determined experimentally? Although resonant excitation and AFM height are consistent, they themselves are not definite evidence for assigning the chirality. Additionally, AFM does not have the lateral resolution to resolve individual nanotubes vs. small bundles.

In EFM sample preparation, what is the purpose for first covering the silica++ substrate with "two layers of sodium cholate by spin coating"? How is it determined that the coating is "two layers"? What happens if this sodium cholate coating is not performed? What is the surfactant/nanotube concentrations of the "dilute" solution of SWCNTs that are subsequently deposited?

For the photoexcitation measurements, what is the power density of the illuminating laser? How quickly do the excited nanotube electrons dissipate? Does the EFM have the time resolution required to capture what occurs immediately following the laser excitation? What would happen if the nanotube, in Figure 3 for instance, is scanned over time?

The discussions on exciton localization and quantum information appear out of place. The work here is focused on resolving charges by EFM, with some evidence for photoexcited electrons. It is unclear how excitons and quantum information are relevant here.

Author's Response to Peer Review Comments:

Please see attached response letter.

16 October 2022

Dr. Editor
Executive Editor, *The Journal of Physical Chemistry Letters*

Dear Dr. Editor,

We would like to thank you and the reviewers for considering our manuscript titled, "Localized Charge on Surfactant Wrapped Single-Walled Carbon Nanotubes" for publication in *The Journal of Physical Chemistry Letters*. We are very pleased that the manuscript received positive assessments, with reviewers agreeing that our work is highly significant and presented in a compelling manner. We extend our gratitude to the reviewers who provided comments; their perspective and insight proved very helpful in the revision of our manuscript. Our responses to all comments and modifications to the manuscript are detailed below:

Reviewer: 1

1. *At the highest level, I'm not sure I understand how the authors confirm that the charged surfactant regions observed on SWCNTs by EFM introduce charges into the SWCNTs. The surfactant clumps show large charge density values on their own, whether or not they are adsorbed to the SWCNTs, so how does observing a charged surfactant clump on a SWCNT translate to knowledge that the observed charges are actually on the SWCNTs (i.e. the SWCNTs are doped) in that same region.*

   We thank the reviewers for their question. We regret that our language appears to be slightly misleading, causing Reviewer 1 to assume that the SWCNTs were being doped by free charges. Any charges that EFM measures before photoexcitation originate from surfactant alone, since bare NTs image neutral (see Figure 2d-f). Further, these are bound charges (from the surfactant) that could be in close proximity to the NT, or physically in contact with the NT. This difference is impossible to tell from EFM. Nonetheless, we are not suggesting that these surfactant related charges are doping the SWCNTs nearby. However, we are suggesting that an electrostatic interaction between the charged surfactant clump and the SWCNT warps the local potential energy of the SWCNT in that region (Figure 4). Thus, when we observe a change in charge in the same region of the surfactant aggregate after photoexcitation, we infer that photoexcited free charge carriers within the SWCNT are trapped in this potential well, which would ultimately change the net charge that EFM measures in that region. If, instead, there was no electrostatic interaction between the surfactant and the SWCNT charge carriers (upon photoexcitation), then the spatial charge profile of the surfactant-SWCNT system that EFM measures would not change after photoexcitation.

   To be more clear that the charges measured by EFM originate from surfactant alone, the following sentence on page 6 of the manuscript was altered: "To confirm that the measured charges were originating from the surfactant and were not associated with the SWCNT, artifacts of the EFM system, ambient conditions, or the Si^{++} substrate, we repeated the measurements with

another ionic surfactant, sodium deoxycholate (DOC), which could be efficiently rinsed off of the SWCNTs."

In order to prevent interpretations that surfactant charge is doping the SWCNTs, we altered the following sentence on page 8 of the manuscript: "EFM measurements demonstrate that surfactant aggregates are charged, however what remains unclear is the extent of the impact these adsorbed charges have on the local energy band structure of the SWCNT."

EFM measurements cannot spatially differentiate between the SWCNT and the surfactant adsorbed to the SWCNT. To account for this point, the following sentence has been altered on pages 8-9 of the manuscript: "For example, as seen in the charge difference image (Figure 3d), photoexcitation resulted in a portion of the surfactant-SWCNT system (i.e. red arrow in Figure 3d and red shaded region in Figs. 3e,f) to become more negative by ~ 14 e. Likewise, another portion of the surfactant-SWCNT system (i.e. purple arrow in Figure 3d and purple shaded region in Figure 3e,f) becomes ~12 e more negative."

In order to more directly answer how observing a charged surfactant clump on a SWCNT translates to knowledge that the observed charge changes are from the SWCNT in that same region, the following sentences were added to the manuscript on page 9: "The EFM-measured charge profile of the surfactant-SWCNT system should not change unless there is an electrostatic interaction between charged surfactant aggregates and photoexcited free charge carriers within the SWCNT. If the surfactant charge is not altering the SWCNT local potential energy, the photoexcited charge carriers would delocalize throughout the length of the nanotube and charge profile should remain the same after photoexcitation."

In order to prevent interpretations that surfactant is doping or "imparting" charge into the SWCNTs, we altered the following sentence on page 12 of the manuscript: "From our results, we infer that regions of charge localization will undoubtably impact SWCNT photophysical properties."

In order to prevent interpretations that surfactant is doping or "introducing" charge to the SWCNTs, we altered the following sentence on page 13 of the manuscript: "In conclusion, sizable charges are interacting with SWCNTs when solubilized with standard ionic surfactants."

2. *Related to this question, I am wondering about how quantitative of a conclusion can be drawn from the EFM measurements related to the SWCNT charge density. The phrasing used in the manuscript (e.g. 'This particular SWCNT has calculated linear charge density values ranging between -160 e/um and 40 e/um…') appears to imply that all locally observed charges for a particular surfactant coated region are assumed to be on the SWCNT. Is this the interpretation? If that is the case, can the authors use these linear charge density values to estimate what this would translate to for the Fermi level of the SWCNT? Or is the interpretation more qualitative than that, i.e. that the observation of locally charged surfactant clumps implies that the SWCNT is charged due to the presence of that local charge density, but that the total number of charges on the SWCNTs cannot be deduced from the measurements*

As a follow-up to the previous question/response, we note that the linear charge densities measured and reported along the SWCNT are simply due to the ionic surfactant only, as a bare SWCNT images as neutral. Therefore, the interpretation of all charge densities reported prior to photoexcitation is that all of the charges are associated with surfactant aggregates and are not associated with the SWCNT. For surfactant coated SWCNTs, we assume that the entire SWCNT

surface is coated with a thin layer of surfactant in addition to having aggregated regions. Linear charge densities are calculated along the lightly coated SWCNT regions due to the 1D line geometry and 2D areal densities are calculated for larger surfactant covered areas. We do not believe the SWCNTs are n or p doped from these adsorbed surfactants and therefore the intrinsic Fermi level of the SWCNT is not substantially changed. However, depending on the polarity of the surfactant charge, photoexcitation results in either free electrons or holes within the SWCNT to be trapped in the potential well estimated to be on the order of hundreds of meV (Figure 4 c-e). Therefore, the nanotube is not being doped (since charge carriers are not being added or removed) but rather free photoexcited carriers within the tube are redistributed spatially due to electrostatic interactions with charged regions. Quantitatively, charge densities as measured by EFM are used to estimate the depth of this electrostatically induced potential well using a 1-D Schrödinger equation.

In order to prevent interpretations that all locally observed charges for a particular surfactant coated region are assumed to be on the SWCNT, and that we don't measure charge from the SWCNT independently of its surfactant coating (pre photoexcitation), we altered the following sentences on page 6 of the manuscript: "This particular SC coated SWCNT has calculated linear charge density values ranging between -160 e/μm and 40 e/μm (or equivalently, -8 and +2 *e* per the diameter of the EFM tip) with the higher magnitudes of charge originating from regions of heavier surfactant coverage. Likewise, EFM measurements of surfactant aggregates away from the SWCNT show both positive and negative charges of similar magnitude to what we observe on the surfactant coated SWCNT (white circle, Figure 2a,b)."

3. *The authors conclude from photoexcitation (PE) experiments that certain surfactant-coated areas of the SWCNTs become more electron- or hole-rich, potentially due to the local band structure in the coated regions forming an exciton localization (or dissociation) site. Looking at Figure 3b and 3c, it looks like there are surfactant clumps off of the SWCNT that also undergo relatively large changes in charge density after PE. How do the authors discern that the SWCNT actually plays a role in these PE experiments? Can the authors compare the spatially probed charge densities before/after PE for the area away from the SWCNT to serve as the control group to compare against? If so, what are the criteria that indicate a statistically significant change above what is observed for surfactant away from the SWCNTs?*

We thank the reviewer for their helpful comment which led us to investigate the photoinduced (PE) charge statistics for each of the 21 background surfactant aggregates shown in Figure 3 and Figure 4/S4. We added a figure to the supporting information, Figure S5, which summarizes this analysis. Ten of the background surfactant aggregates change in charge by less than 1 *e*. Only one surfactant aggregate changes by more than 4 *e*. Thus, the changes in charge exhibited by the surfactant clumps in the AFM image background are on average significantly smaller than the changes in the localized charge measured along the SWCNT (>10-14 e). We note that the size of the aggregate does not have any correlation with charge magnitude or net charge change. For example, the largest aggregate in the background is labeled number 13 in Figure S5 a-c. That aggregate measures ~0 e with the smallest recorded change in charge after PE. On the other hand, a very small aggregate, labeled number 3 in Figure S5 d-f, measures ~-3 *e* before PE, and decreases by nearly 5 *e* after photoexcitation.

While we are photoexciting at 561 nm (E_{22} transition in (6,5) SWCNTs) intending to only excite the SWCNT, our data also shows changes in charge for surfactant aggregates in the background of our images, away from the longer SWCNTs in the image. One explanation for the changes in charge is that with EFM and AFM, our images cannot differentiate between a surfactant

aggregate in the background and a surfactant aggregate covering a small SWCNT fragment. Surfactant aggregates numbers 6 and 13 (Figure S5a), and numbers 5 and 8 (Figure S5b) are large enough to host a SWCNT fragment. If we assume there are no "hidden" SWCNT fragments, an alternative mechanism for the small change in charge a heating mechanism during the 10 minute period that the 50 mW laser is illuminating the sample. If water is intercalated within the surfactant aggregates, the laser heat could cause a rearrangement of the surfactant and the compensating cations which could alter the net charge of the aggregate. To address this point in the main text, we have added the following sentence on page 10: "There are slight changes in background surfactant charge after photoexcitation, potentially due to small SWCNT fragments hidden by the aggregate and/or heat induced surfactant rearrangements during photoexcitation (See Figure S5 for background surfactant aggregate charge statistics). Ultimately, the changes in charge (50% of aggregates changing by less than 1 *e*, 90% of aggregates changing by less than 3 *e*) are much smaller than the photoinduced changes at the localization sites measured along the SWCNT which vary by magnitudes of ~10 to 20 *e*."

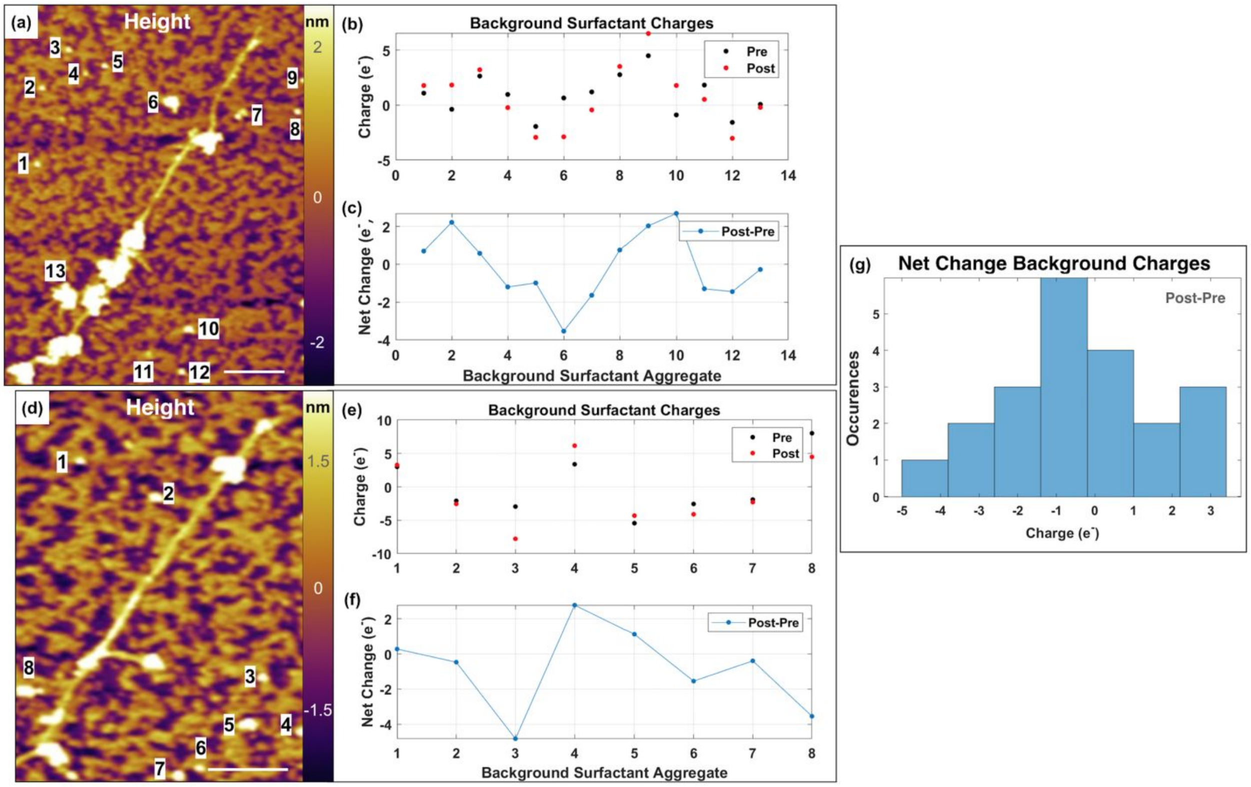

Figure S5. (a) Topographic AFM image (Figure 3) with surfactant aggregates away from the SWCNT numbered 1-13. (b) Calculated charge e pre (black) and post (red) photoexcitation plotted for surfactant aggregates 1-13. (c) Plotted charge difference (post PE minus pre PE) for surfactant aggregates 1-13. (d) Topographic AFM image (Figure 4 and Figure S4) with surfactant aggregates away from the SWCNT numbered 1-8. (e) Calculated charge e pre (black) and post (red) photoexcitation plotted for surfactant aggregates 1-8. (f) Plotted charge difference (post PE minus pre PE) for surfactant aggregates 1-8. (g) Histogram displaying the net change in charge for 21 background surfactant aggregates.

4. *Water, which has a very high dielectric constant, is known to fill the endohedral space within SWCNTs, which could play an important role in the charge densities observed in the current experiments. It would also seem feasible that the large surfactant clumps that the authors observe in their experiments may have water molecules intercalated within the clumps. Can the authors comment on the degree to which water may impact their results? I also did not see any annealing*

step(s) discussed in the Methods. Could the authors compare the EFM-measured charge density profiles before and after an anneal step that would potentially remove some/all of the water molecules within the SWCNTs and within the surfactant clumps?

The reviewers are correct that we did not perform any annealing steps in our methods. Our EFM measurements are performed under ambient conditions and additionally, our SWCNT samples are prepared in water. Therefore, we agree with the reviewers that water is present during our measurements, possibly intercalated within surfactant aggregates and within the SWCNT itself. However, regardless of the possible water-surfactant interaction, we believe that it is highly unlikely that water filling the endohedral space contributes to the charges we measure along the SWCNTs. For our control experiment (Figure 2 d-f) we demonstrated that the charges we measure must arise from the surfactant, not the substrate, SWCNTs themselves, or the environment. In that experiment, DOC-SWCNTs (sodium deoxycholate surfactant) spun coat on the silica^{++} substrate were efficiently rinsed with isopropyl alcohol to remove the DOC surfactant. EFM measurements revealed a neutral charge along the bare SWCNT. These SWCNTs were not annealed and water was likely present, remaining after the IPA soaks or arising from the air/humidity. With water present, EFM measurements showed neutral charge, and therefore we suggest that surfactant is the major contributor to measured charges.

To address this point in the main text, we have modified the following sentence on page 6: “To confirm that the measured charges were originating from the surfactant and were not associated with the SWCNT, artifacts of the EFM system, ambient conditions, or the Si^{++} substrate, we repeated the measurements with another ionic surfactant, sodium deoxycholate (DOC), which could be efficiently rinsed off of the SWCNTs.”

Even so, we performed the annealing experiment suggested by Reviewer 1 following the same sample preparation for the SC-SWCNT system up to the annealing step. After the sample was prepared, it was annealed at 110ºC for 1 hour. While the sample was in the oven, the EFM chamber was filled with nitrogen for 1 hour. The sample was subsequently transferred to the EFM chamber to cool under nitrogen for 20 minutes. EFM measurements were performed under constant nitrogen flow. Charge density profiles are similar to what was measured on samples that were not annealed, supporting our conclusion that charges measured originate from surfactant, not water. Figure S3 was added to the supporting information. Additionally, the following sentence was added to page 8: “To ensure the measured charge was not impacted by the presence of water, an additional control experiment was performed. A sample of SC-SWCNT was annealed prior to imaging under nitrogen flow. EFM signals (Figure S3) acquired after heating resulted in similar charge magnitudes as unheated samples, thus confirming that any water molecules intercalated within the tubes or surfactant aggregates (in unheated samples) do not lead to any measurable charge screening.” The annealing procedure was added on page 14 of the manuscript under *Sample preparation EFM experiments (SC-SWCNT system)*:”For the annealing control procedure only, the sample was heated at 110ºC for 1 hour while the EFM chamber was filled with nitrogen. The sample was subsequently transferred to the EFM chamber to cool under nitrogen for 20 minutes. EFM measurements were performed under constant nitrogen flow.”

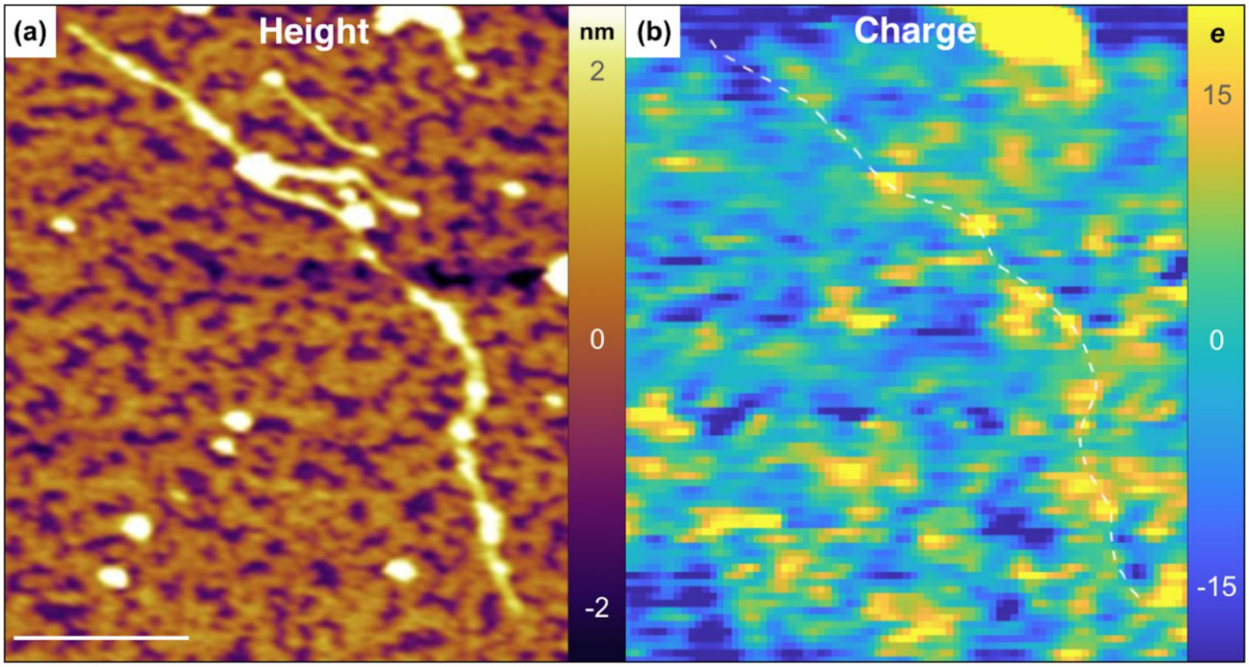

Figure S3. EFM images of (a) topography and (b) charge profile for a SWCNT coated with sodium cholate surfactant after annealing. Scale bar is 500 nm.

5. *It would be helpful for the authors to explicitly state early on the sign convention used in the charge profile images, line scans, etc. When I saw that the charge densities are provided in units of electrons, my initial interpretation (taking Figure 3 as an example) is that positive areas correspond to positive numbers of electrons (i.e. negatively charged due to electron accumulation), while negative areas correspond to regions where electrons have been removed (i.e. positively charged). It was only after reading the PE description on page 9 where the authors indicate that particular regions in Figures 3f with negative signs 'become more negative' that the authors are defining charge density in the opposite fashion of my original interpretation.*

   We thank the reviewer for their feedback on clearly stating the sign convention. The following sentence has been added to the main text on page 6. "The sign convention for charge densities used in this study is as follows: positive areas (yellow) corresponds to positive charge and negative areas (blue) correspond to negative charge."

6. *Does the majority carrier in the silicon wafer used as the sample substrate play any role in the typical carrier type observed in the SWCNTs? In the current experiments, the authors use Si^{++} and it seems like typical pre-PE measurements indicated hole accumulation on the SWCNTs (Fig. 3e, Fig. 4b). Have the authors performed measurements on n-type Si to see if the primary carrier sign observed by EFM flips to be electron-dominated?*

   The reviewer poses an interesting question. We don't believe the majority carrier in the silicon wafer used as the sample substrate plays a significant role in the typical carrier type observed in the SWCNTs. Absent photoexcitation, the highly conductive Si just serves as a counter electrode, i.e. there is no mechanism for bound charges to leave the surfactant. Indeed, as shown in Figure S2, the sign of the surfactant charge is evenly distributed about zero, as expected. It is possible

that the silicon^{++} wafer could accept free electrons or holes from the SWCNTs after photoexcitation; however, this was not observed as overall the NTs remained neutral after photoexcitation (Figure 3f). Thus, we conclude that under our imaging conditions, the silicon^{++} wafer is not affecting the measurements.

7. *Does the charge of the surfactant head group play a role in the predominant carrier type observed in the SWCNTs? Since the authors observe predominantly hole rich SWCNTs for these negatively charged surfactants, could the authors compare these measurements to a positively charged surfactant (e.g. CTAB) to tease out the impact of the head group charge?*

   The charge of the surfactant head group does not seem to play a role in the predominate carrier type observed in the SWCNTs. In a similar response to point 6, in Figure S2a we show the sign of the surfactant charge on the nanotube and in the background is evenly distributed. Although sodium cholate has a negatively charged head group, we observe a similar distribution of positively charged surfactant aggregates as we do negatively charged surfactant aggregates. We expect that changing to a cationic surfactant, such as CTAB, will result in a similar distribution of charge. However, the process of solubilizing NTs in CTAB and obtaining long NTs for viable AFM and EFM measurements is a non-trivial undertaking and thus beyond the scope of this current study.

Reviewer: 2

Comments:

1. *Figure 2. How is the DOC surfactant removed with IPA? This is important to understand this control, but the detail of the procedure is not provided.*

   We thank the reviewers for pointing out this missing procedure. We added a procedure detailing the suspension of SWCNTs in sodium deoxycholate surfactant on page 14 of the manuscript: "Suspensions of CoMoCAT-manufactured SWCNTs were prepared at a concentration of 1 mg/mL in 5 mg/mL sodium deoxycholate (DOC) in nanopure H_2O (Barnstead Micropure, Thermo Fisher Scientific). The DOC-SWCNT solution was tip sonicated (Branson 450 Sonifier, 16 W, 20 kHz, constant) for 1 h while immersed in an ice bath. The resulting dispersion was ultracentrifuged at 45560g (Beckman Optima L-90 K, SW-41 rotor) for 4 hours, and the supernatant was filtered using a 5 µm syringe filter (Millipore Millex-SV). SWCNTs with lengths of ~ 1 µm were routinely observed during EFM data collection. Charges measured from the DOC surfactant-SWCNT system were comparable to the SC surfactant-SWCNT system before rinsing."

   We added a procedure detailing EFM sample preparation for the DOC-SWCNTs on pages 14-15 of the manuscript: "A silica^{++} substrate (Silicon Valley Microelectronics) with a few nm thick oxide layer was first covered in two depositions of DOC by spin coating (Speedline Technologies) 30 µL of 1% wt. DOC in nanopure H_2O (Barnstead Micropure, Thermo Fisher Scientific) for 60 sec at 3000 rpm (and repeating) to help with SWCNT adhesion. A 2x dilution of the DOC SWCNT solution was bath sonicated for 1 minute, then 50 µL was spin coated for 60 seconds at 3000rpm."

   We added a procedure detailing the removal of DOC with isopropyl alcohol on page 15 of the manuscript: "The DOC-SWCNT covered silica^{++} substrate was soaked in isopropyl alcohol

(Thermo Fisher Scientific) for 5 minutes. After removal from the IPA soak, it was further rinsed by lightly squirting IPA onto the substrate. The substrate was promptly dried under a stream of N_2 gas and then imaged. This removal process was repeated (~10 rounds) until AFM topographic images showed no remaining surfactant (verified visually and via line cuts of the SWCNT height)."

2. *Figure 3. In one of the surfactant aggregates, the photoexcitation is shown to deplete the electrons, but this phenomenon is not observed in other regions of the same nanotube. Are there other factors at play here? What are the statistics in observing such photoexcitation induced depletion of electrons?*

The surfactant aggregates in Figure 3 are positively charged before photoexcitation. The charged surfactant aggregates interact with photoexcited free electrons in the SWCNT, via Coulomb attraction, leading to their localization. Thus, after photoexcitation, EFM measures a decrease in charge in that region (blue color in Figure 3d or negative peaks in Figure 3f). The net charge of the nanotube shown in Figure 3 changed by only 0.8 *e*, supporting our claim that photoexcited charges are compensated throughout the SWCNT. Figure 3f plots both the electron localization sites (under positively charged surfactants) and hole compensation sites. In Figure 3d (difference image), the photoexcitation shows an area of electron depletion (yellow coloring). Reviewer 2 noticed that this phenomenon is not observed in other regions of the same nanotube displayed in Figure 3d. While it is true that one particular region of Figure 3d visually stands out, there are many other regions on this SWCNT that exhibit electron depletion. It is easier to see electron depletion in Figure 3f. Each of the "positive" peaks correspond to regions of electron depletion due to hole compensation. Statistically, with charge compensation, any trapped electron will have a hole compensated elsewhere along the SWCNT. However, it is important to note that our measurements cannot definitively conclude precisely where a trapped electron's hole is located.

3. *How is the (6,5) chirality determined experimentally? Although resonant excitation and AFM height are consistent, they themselves are not definite evidence for assigning the chirality. Additionally, AFM does not have the lateral resolution to resolve individual nanotubes vs. small nanotube bundles.*

We agree with the reviewer that we cannot definitively state that the chirality is exclusively (6,5); however, our sample preparation steps strongly suggest that our sample is dominated by the presence of (6,5) SWCNTs. The raw CoMoCAT carbon nanotube starting material we use contains 90% (6,5) chirality. Upon the addition of sodium cholate surfactant, the larger diameter carbon nanotubes crash out because they are not efficiently coated with the surfactant (which can be confirmed upon the acquisition of an absorbance spectrum). Ultimately, this increases the percentage of (6,5) SWCNTs in the remaining dispersion. Based on the prevalence of (6,5) SWCNTs in our sample, we assumed the (6,5) chirality for the charge numerical modeling which was based on our experimental results. It is important to note that (7,5) SWCNTs may also be present in reasonable amounts in the final sample. While we cannot differentiate between (6,5) and (7,5) SWCNTs from topographic images due to their similar diameters, we are confident that the potential presence of (7,5) SWCNTs does not alter our analysis. Although resonant E_{22} excitation for (7,5) SWCNTs requires ~645 nm irradiation, (7,5) SWCNTs can still photoluminesce upon excitation at 561 nm, indicating that exciton creation is efficient at this wavelength.

To ensure we address this point directly, we added two sentences on pages 7-8 of the manuscript: "AFM and EFM measurements alone cannot experimentally distinguish between certain small diameter chiralities of SWCNTs. However, CoMoCAT carbon nanotubes are 90% (6,5) chirality as manufactured. Based on that prevalence, we assume the (6,5) chirality for SWCNTs in various experimental and theoretical analyses throughout this study."

We agree with reviewers that the AFM does not have the lateral resolution to resolve individual SWCNTs versus small bundles of SWCNTs. Despite the use of surfactant to prevent aggregation of SWCNTs into bundles, and despite centrifugation and bath sonication, we still find bundles during our AFM/EFM imaging. However, the lateral resolution of the AFM is not a common metric used to differentiate bundles from individual nanotubes. Based on our experience imaging bundles, we believe the SWCNTs we present in this study are individual SWCNTs. To confidently identify individual SWCNTs, line cuts are made along the length of the SWCNT present in AFM topographic images. As is shown in Figure 2(c,f), the height trace perpendicular to the nanotube produces a single, uniformly distributed peak. For a small SWCNT bundle, we would expect the emergence of a shoulder or secondary peak from a single line cut. Additionally, our sample preparation methods would not likely result in perfectly bundled nanotubes that visually resemble a single nanotube on the substrate. The centrifugation procedure removes large bundles from the supernatant. Any remaining bundles are disrupted via bath sonication, a technique we always use before spin coating SWCNTs onto the substrate. Lastly, with the spin coating deposition method, it is highly unlikely for two or a few nanotubes to land on the substrate in a manner where they are perfectly woven together throughout their entire length. For a bundle after spin coating at 3000 rpm, it is more probable that nanotube ends would separate from the bundle, which would be easily detected with AFM.

4. *In EFM sample preparation, what is the purpose for first covering the silica ++ substrate with "two layers of sodium cholate by spin coating"? How is it determined that the coating is "two layers"? What happens if this sodium cholate coating is not performed? What is the surfactant/nanotube concentrations of the "dilute" solution of SWCNTs that are subsequently deposited?*

We thank the reviewers for highlighting this point of confusion. We have changed our imprecise language from "layers" to "depositions" instead on page 14 of the manuscript: "A silica^{++} substrate (Silicon Valley Microelectronics) with a few nm thick oxide layer was first covered in two depositions of sodium cholate by spin coating (Speedline Technologies) 30μL of 1%wt sodium cholate solution for 60 sec at 3000 rpm (and repeating) to help with SWCNT adhesion." If the sodium cholate depositions are not performed, the SC-SWCNT solution will not sufficiently adhere to the substrate.

We have also clarified our reference to the "dilute" solution of SC-SWCNTs that is subsequently deposited. The following sentence was changed on page 14 of the manuscript: "A 2x dilution of the shear force mixed SWCNTs dispersed in sodium cholate was bath sonicated for 1 minute, then 50μL was spin-coated for 60 seconds at 3000rpm." It is important to note that, although the surfactant deposition prior to addition of SWCNTs helps the adhesion process, not every SWCNT will stick during spin coating. As a result, it is difficult to ascertain the exact concentration of SWCNTs on the substrate surface.

5. *For the photoexcitation measurements, what is the power density of the illuminating laser? How quickly do the excited nanotube electrons dissipate? Does the EFM have the time resolution required to capture what occurs immediately following the laser excitation? What would happen if the nanotube, in Figure 3 for instance, is scanned over time?*

For the photoexcitation measurements, the power density of the illuminating laser is estimated to be 1.6 W/cm^2. We modified an existing sentence on page 4 of the supporting information to include the power density: "Our samples were photoexcited at a grazing angle with 561 nm laser light (CW laser) (Coherent OBIS) at an approximate intensity of 1.6 W/cm^2."

For optimal imaging, the acquisition time for one image is ~ 11 minutes. We cannot excite the SWCNTs during image acquisition due to the laser interfering with image quality. Instead, we collect EFM measurements following a 10 minute illumination period. During the EFM scan after photoexcitation (laser off), the changes in charge remain for the duration of the 11+ minute scan. Beyond the 11 minute EFM scan immediately following photoexcitation, the charges will likely not remain trapped indefinitely, but slowly decay back to the original charge state. For example, for CdSe NCs on graphite, recovery of the charge state after photoexcitation took dozens of minutes to hours (J. Phys. Chem. B 2001, **105**, 1725-1733). Since each NT would have to be studied individually, over a period of hours, obtaining charge recovery data post photoexcitation on enough NTs to have meaningful statistics would be a significant undertaking. While we agree that Reviewer 2 poses an interesting question, practically this experiment would be excellent for future EFM studies.

6. *The discussions on exciton localization and quantum information science appear out of place. The work here is focused on resolving charges by EFM with some evidence for photoexcited electrons. It is unclear how quantum information are relevant here.*

Our work provides evidence of localized charges along SWCNTs due to electrostatic interactions with charged surfactant aggregates on the NT surface. Ultimately, we infer that this charge localization will lead to exciton traps upon photoexcitation and thus create 'quantum dot' like single photon emission. While we agree with the reviewers that the focus of this manuscript is quantifying charges on NT surface via EFM, our findings indicate that the measured large magnitude of charges can significantly warp the local potential energy of the NT similar to sp^3 defects which have demonstrated to be excellent single photon emitters for QIS.[1, 2]

The relevancy to quantum information science is that indistinguishable single photons in the telecom band are needed as encoded qubits to carry information and a SWCNT system with localized excitons via controlled deposition of surfactant charges or applied electric fields in theory could potentially be a robust near-IR single photon source.

The following sentence was altered on page 3 of the manuscript to introduce this connection between SWCNTs and QIS: "Single-walled carbon nanotubes (SWCNTs) are quasi-one-dimensional graphitic materials with unique size-dependent optical and electronic properties, making them potentially suitable for a variety of applications, including water oxidation, energy storage, photovoltaics, field-effect transistors, chemical and biological sensors, and quantum technology."
The following sentence was altered on page 4 of the manuscript to make this connection clearer: "For example, counterions adsorbed to the nanotube surface lead to localized charges along the SWCNT which can trap excitons upon photoexcitation and thus lead to single photon emission characteristics."
The following sentence was altered on page 4 of the manuscript to introduce the connection between our work, the work of SWCNT community in relation to QIS: "Our findings indicate that surfactant charge impurities significantly warp the local potential energy of the SWCNT,

similar to *sp*^{3} defects which have demonstrated to be excellent single photon emitters for quantum information science."

A number of other changes were made to improve the overall clarity of the manuscript and to further remove any misleading language. These edits are shown in the tracked changes word document.

We thank you for the opportunity to revise our manuscript and hope that it is in an acceptable form for publication.

Sincerely,

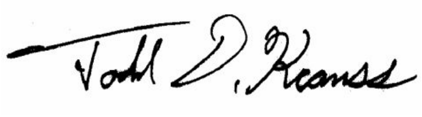

Dr. Todd D. Krauss
University of Rochester
585-275-5093
krauss@chem.rochester.edu

(1) Ma, X.; Hartmann, N. F.; Baldwin, J. K. S.; Doorn, S. K.; Htoon, H. Room-Temperature Single-Photon Generation from Solitary Dopants of Carbon Nanotubes. *Nat. Nanotechnol.* **2015**, *10*, 671-675
(2) He, X.; Hartmann, N. F.; Ma, X.; Kim, Y.; Ihly, R.; Blackburn, J. L.; Gao, W.; Kono, J.; Yomogida, Y.; Hirano, A.; Tanaka, T.; Kataura, H.; Htoon, H.; Doorn, S. K. Tunable Room-Temperature Single-Photon Emission at Telecom Wavelengths from Sp3 Defects in Carbon Nanotubes. *Nat. Photon.* **2017**, *11*, 577-582

jz-2022-026509.R2

Name: Peer Review Information for "Localized Charge on Surfactant Wrapped Single-Walled Carbon Nanotubes"

Second Round of Reviewer Comments

Reviewer: 2

Comments to the Author

The authors have addressed my previous concerns. In the revision, the statement that CoMoCAT carbon nanotubes are 90% (6,5) chirality is unlikely correct.

Reviewer: 1

Comments to the Author

The authors have addressed reviewer comments appropriately.

Author's Response to Peer Review Comments:

Please see attached response to decision letter.

2 November 2022

Dr. Editor
Executive Editor, *The Journal of Physical Chemistry Letters*

Dear Dr. Editor,

We would like to thank you and the referees for reviewing our revised manuscript titled, "Localized Charge on Surfactant Wrapped Single-Walled Carbon Nanotubes" for publication in *The Journal of Physical Chemistry Letters*. We are very pleased that the revised manuscript received very positive assessments. Our responses to Reviewer 2's comment and the corresponding modification to the manuscript is detailed below:

Reviewer: 2

> *The authors have addressed my previous concerns. In the revision, the statement that CoMoCAT carbon nanotubes are 90% (6,5) chirality is unlikely correct.*
>
> We regret the confusion and have addressed Reviewer 2's comment in the manuscript on pages 7-8 by changing "90%" to "majority" and making clear that the predominance of (6,5) is only after our processing. In addition, we added a figure of NT absorbance to the SI that shows that after our processing a majority of NTs are (6,5).
>
> Changes to text on pages 7-8: "However, CoMoCAT carbon nanotubes are majority (6,5) chirality after suspension (Figure S3)."

We thank you for the opportunity to revise our manuscript and hope that it is in an acceptable form for publication.

Sincerely,

Dr. Todd D. Krauss
University of Rochester
585-275-5093
krauss@chem.rochester.edu
